# Evaluation of Genome Sequencing Quality in Selected Plant Species Using Expressed Sequence Tags

**DOI:** 10.1371/journal.pone.0069890

**Published:** 2013-07-29

**Authors:** Lingfei Shangguan, Jian Han, Emrul Kayesh, Xin Sun, Changqing Zhang, Tariq Pervaiz, Xicheng Wen, Jinggui Fang

**Affiliations:** 1 College of Horticulture, Nanjing Agricultural University, Nanjing City, Jiangsu Province, China; 2 College of Horticulture, Jinling Institute of Technology, Nanjing City, Jiangsu Province, China; UCLA-DOE Institute for Genomics and Proteomics, United States of America

## Abstract

**Background:**

With the completion of genome sequencing projects for more than 30 plant species, large volumes of genome sequences have been produced and stored in online databases. Advancements in sequencing technologies have reduced the cost and time of whole genome sequencing enabling more and more plants to be subjected to genome sequencing. Despite this, genome sequence qualities of multiple plants have not been evaluated.

**Methodology/Principal Finding:**

Integrity and accuracy were calculated to evaluate the genome sequence quality of 32 plants. The integrity of a genome sequence is presented by the ratio of chromosome size and genome size (or between scaffold size and genome size), which ranged from 55.31% to nearly 100%. The accuracy of genome sequence was presented by the ratio between matched EST and selected ESTs where 52.93% ∼ 98.28% and 89.02% ∼ 98.85% of the randomly selected clean ESTs could be mapped to chromosome and scaffold sequences, respectively. According to the integrity, accuracy and other analysis of each plant species, thirteen plant species were divided into four levels. *Arabidopsis thaliana*, *Oryza sativa* and *Zea mays* had the highest quality, followed by *Brachypodium distachyon*, *Populus trichocarpa*, *Vitis vinifera* and *Glycine max, Sorghum bicolor*, *Solanum lycopersicum* and *Fragaria vesca*, and *Lotus japonicus*, *Medicago truncatula* and *Malus* × *domestica* in that order. Assembling the scaffold sequences into chromosome sequences should be the primary task for the remaining nineteen species. Low GC content and repeat DNA influences genome sequence assembly.

**Conclusion:**

The quality of plant genome sequences was found to be lower than envisaged and thus the rapid development of genome sequencing projects as well as research on bioinformatics tools and the algorithms of genome sequence assembly should provide increased processing and correction of genome sequences that have already been published.

## Introduction

Whole genome sequencing is a technique that can determine complete DNA sequences of organisms ranging from chromosomal, mitochondrial, and chloroplast DNA (in plant). So far, DNA sequencing technology has undergone three stages of development, namely the Sanger, next-generation, and third-generation sequencing methodologies.

Chain-terminating dideoxynucleotides triphosphates (“Sanger method”) has been used for more than 30 years [1], [2]. After the Sanger method, another DNA sequencing method known as “Chemical sequencing” which is based on chemical modification of DNA and subsequent cleavage at specific bases was developed [3]. After that, three “next-generation sequencing (NGS)” platforms including the Roche/454 Genome Sequencer (http://www.454.com) [4], Illumina/Solexa Genome Analyzer II (http://www.Illumina.com) [5], [6] and Applied Biosystems SOLiD System (http://www.solid.appliedbiosystems.com) [7] were introduced and made commercially available. These platforms have been widely used in many genome sequencing projects. Three additional platforms namely the HeliScope™ Single Molecule Sequencer [8], [9], Pacific Biosmart SMRT (single-molecule real time) [10], and Oxford Company Nanopore sequencing technology [11] were also released and aptly referred to as the “third-generation sequencing (TGS)” technologies [12] and are characterized by lower cost, higher throughput, much longer sequencing fragments and faster speed.

High cost and low throughput are two key limitations plaguing whole genome sequencing. The development of NGS and TGS technologies has greatly increased the throughput and drastically reduced the cost of sequence generation. According to the report of National Human Genome Research Institute (NHGRI), the cost per Mb DNA sequence was $5,292.39 in 2001, $974.16 in 2005, even $0.09 in 2012, while the cost per genome sequence was $95,263,072, $9,408,739 and $7,666 in 2001, 2007 and 2012, respectively [13]. The NGS platforms provided ideal sequencing coverage, as depicted by Sanger-based sequencing (average read length = 500–600 bases), 6-fold coverage; 454 sequencing (average read length = 300–400 bases), 10-fold coverage; and Illumina and SOLiD sequencing (average read length = 50–100 bases), 30-fold coverage. Commercialization of full genome sequencing is growing rapidly aided by the tremendous progress in sequencing technology.

Availability of DNA sequencing technologies directly promotes the development of genome sequencing projects. The first plant subjected to whole-genome sequencing was *Arabidopsis thaliana,* in 2000 [14]. This was followed by completion of genome sequencing projects of several important plant species for instance rice, poplar, grape, maize and tomato in 2002, 2006, 2007, 2009 and 2012 respectively [15]–[20]. To date more than 30 plant genome sequencing projects have been finished (http://www.mgrc.com.my/list_eukaryotic_genomes.shtml) and many more plant genomes are at different stages of sequencing and assembly (http://www.ncbi.nlm.nih.gov/genomes/PLANTS/PlantList.html).

Most genome sequencing data is stored in online databases, such as NCBI (http://www.ncbi.nlm.nih.gov/), TAIR (http://www.arabidopsis.org/), and Genoscope (http://www.genoscope.cns.fr/externe/GenomeBrowser/Vitis/). These databases are available to the public and researchers who can freely download the chromosome sequences, scaffold sequences, nucleotide sequence and protein sequence of predicted gene, and the annotation of homologous genes. Most plant biologists are concerned with the reliability and utilization of data from whole genome sequences. High quality of genome sequence data especially that of chromosome sequences, forms important basis of model genomics, molecular biology, breeding science, structural biology and molecular evolution. Unlike *Arabidopsis* and rice, which were sequenced by the Sanger’s method using a BAC-by-BAC approach, and have their genome sequencing completed, the sequences of the rest of the plants are being drafted in a greater or lesser stage of completion. Some complete or gold standard genomes still contain gaps in their sequences corresponding to highly repetitive sequences which are recalcitrant to sequencing and assembly methods [21]. If there are too many assemble fragments or error sequences, it will negatively influence on the downstream analysis, even cause a wrong biological conclusion [22].

Expressed sequence tags (ESTs) are partial sequences of complementary DNA (cDNA) clones from both ends of expressed gene fragments [23], and have been widely used for large-scale expression analysis, mapping genes to chromosomes as ‘sequence-tagged sites’ (STSs), elucidating phylogenetic relationships [23]–[28], and mapping ESTs to chromosome sequences. By July 1, 2012, 73,360,923 ESTs had been deposited in the dbEST of NCBI (http://www.ncbi.nlm.nih.gov/dbEST/dbEST_summary.html), and the leading five plants in terms of ESTs reported were *Arabidopsis thaliana* (1,529,700), *Glycine max* (1,461,624), *Triticum aestivum* (1,286,173), *Oryza sativa* (1,252,989), and *Panicum virgatum* (720,590). To map EST sequences to a genome and promote research advances in EST alignment on genome, est_genome, dds/gap2, sim4, Spidey, GeneSeqer, MGAlign, est2genome (a software of EMBOSS packet), GMAP, and ESTmapper softwares [29]–[38] have also been introduced.

These EST sequences, public genome sequences and bioinformatics tools provide a possible way to evaluate the quality of multiple plant genome sequencing projects through mapping the EST sequences to the chromosome/scaffold sequences. Although ESTs could be used in the genome coverage test in a genome sequencing project, there was no comparative research on genome sequencing projects in multiple plant species. For this study, bulk data containing chromosome sequences, scaffold sequences, annotation of homologous gene, and EST sequences for thirty-two plants were downloaded from the databases and integrity and accuracy calculated then used to evaluate the qualities of genome sequence in 32 plant species. The influence of the GC content and three kinds of repeat sequence (interspersed repeat sequence, low complexity sequence and simple repeat sequence) on the genome sequence quality was analyzed and two examples used to show the impacts of low quality of genome sequence in downstream studies.

## Materials and Methods

### Sequence Retrieval and Software Preparation

The plant EST sequences in FASTA format and the lists of GenBank accessions of the ESTs were downloaded from dbEST at the NCBI GenBank FTP site (ftp://ftp.ncbi.nih.gov/genbank/) [39]. The plant genome sequences (chromosome or scaffold sequences), predicted gene sequences, chloroplast and mitochondrial sequences were downloaded from several databases, including the NCBI Genome FTP site (ftp://ftp.ncbi.nlm.nih.gov/genomes/), TAIR FTP site (ftp://ftp.arabidopsis.org/home/tair/Sequences/), Genome Database for Rosaceae (http://www.rosaceae.org/), Genoscope database (http://www.genoscope.cns.fr/externe/GenomeBrowser/Vitis/), Database for comparative plant genomics (http://www.plantgdb.org/), and Phytozome FTP site (ftp://ftp.jgi-psf.org/pub/JGI_data/phytozome/v8.0/) [40]–[44]. The homologous gene annotation files for plants were downloaded from Genoscope (http://www.genoscope.cns.fr/externe/GenomeBrowser/Vitis/), TAIR (http://www.arabidopsis.org/), OrthoInspector (http://lbgi.igbmc.fr/orthoinspector/), and Rice Genome Annotation Project database (http://rice.plantbiology.msu.edu/) [41], [45], [46]. The 16S rRNA sequence dataset was downloaded from the Ribosomal Database Project (RDP) (http://rdp.cme.msu.edu/download/release10_27_unaligned.fa.gz) and the bacterial genome downloaded from the NCBI Genome FTP site (ftp://ftp.ncbi.nlm.nih.gov/genomes/Bacteria/) [40], [47].

The RanEST.pl script was designed to randomly select the GenBank accession from the lists of each plant. The ESTFinder was downloaded from NAU website (http://genomics.njau.edu.cn/software/ESTFinder/) and used to retrieve the bulk EST sequences from local dbEST [48]. The ctrans program was downloaded from NAU website (http://www.njau.edu.cn/down/ctrans/), and used in vector and adaptor sequence clipping [49]. The BLAST program was downloaded from the NCBI FTP site (ftp://ftp.ncbi.nlm.nih.gov/blast/executables/blast/LATEST/, version 2.2.27), and used in 16S rRNA sequence, bacterial sequence, chloroplast DNA and mitochondrial DNA sequence removing [50]. EMBOSS (European Molecular Biology Open Software Suite) program package was downloaded from the emboss public FTP site (ftp://emboss.open-bio.org/pub/EMBOSS/, version 6.40), and installed in an iMac computer [37]. The est2genome program from the EMBOSS program package was used to map the EST sequence to the genome (chromosome/scaffold) sequences. The GCcontent.pl script was designed to calculate the GC content in chromosome sequences. The RepeatMasker program was downloaded from the RepeatMasker homepage (http://www.repeatmasker.org/, version 3.3.0), and used to analyze the repeat DNA distribution in chromosome sequences. The NCBI batch web Conserved-Domains Search tool (http://www.ncbi.nlm.nih.gov/Structure/bwrpsb/bwrpsb.cgi) was used to identify the conserved domain in protein sequence [51].

### Random ESTs Retrieval Steps

There are four steps in random EST sequence retrieval:

- The RanEST.pl script was used in the first round for random EST sequence selection. The selection process followed the following parameters: if the total number of ESTs stored in dbEST >10,000, 50% of total ESTs were selected while if the total number of ESTs stored in dbEST <10,000, 100% of total ESTs were selected for the sequence analysis.
- The ctrans and BLAST programs were used in the removal of vector, adaptor, 16S rRNA, bacterial, chloroplast DNA and mitochondrial DNA sequences in the randomly selected ESTs, thus producing the clean EST sequences.
- The RanEST.pl script was used in the second round of random EST sequence selection from the clean EST sequences. The selection process was such that if the total number of ESTs stored in dbEST >100,000, 1% of total ESTs were selected from the clean ESTs for the EST-Genome mapping (for example, the number of ESTs in the dbEST was 1,529,700, 15,297 clean ESTs were selected for the EST-Genome mapping); if the total number of ESTs stored in dbEST <100,000 and >10,000, 10% of total ESTs were selected from the clean ESTs for the EST-Genome mapping; if the total number of ESTs stored in dbEST <10,000, 100% of total ESTs were selected from the clean ESTs for the EST-Genome mapping.
- Finally, the randomly selected clean EST sequences were downloaded *via* the NCBI Batch Entrez tool (http://www.ncbi.nlm.nih.gov/sites/batchentrez) according to the GenBank accession list files.

### ESTs Mapping to the Chromosome/scaffold Sequences

Randomly selected EST sequences were aligned into chromosome/scaffold sequences using the est2genome program. For chromosome and scaffold sequence statistics, the total number of matched EST sequences in every 0.1 Mb (100,000 bp) segments of the chromosome and the total number of matched EST sequences in all scaffold sequences were determined respectively.

### Calculation of GC Content and Repeat DNA Sequence

GCcontent.pl script was used to calculate the GC content of the whole chromosome sequence or several special regions of the chromosome while the RepeatMasker program was used to detect the repeat DNA sequence distribution in each chromosome.

### Gene Cluster Identification in Six Plants

Ten contiguity gene models were randomly selected in *Arabidopsis thaliana*, and screened for homology in the other five plant species (*Brachypodium distachyon*, *Oryza sativa*, *Populus trichocarpa*, *Vitis vinifera*, and *Zea mays*), using the BLAST tool, OrthoInspector, TAIR, Rice Genome Annotation Project information. If one gene could not be identified in any other species, the *Arabidopsis* gene model was dropped, and the next contiguous gene in *Arabidopsis* was used to identify the homologous genes in other plant species. This was done until ten contiguous genes and their homologous genes were identified in the six selected plant species.

### Detection of Gene Multi-copy

The gene model was downloaded from TAIR database and used as a query to search plant gene sequences in three plant species through BLAST. Alignment parameters were scores >200 and E-value >1×10^−50^. NCBI Batch CD-Search Tool, the information from TAIR, Genoscope, OrthoInpsector, and Rice Genome Annotation Project were used to confirm the gene multi-copy detection results.

## Results

### Ratio of Chromosome Size and Whole Genome Size (or between Scaffold Size and Whole Genome Size) in 32 Plants

The NGS platform can produce a large number of short reads (25–70 bp), which can be assembled into contigs sequences using sequence overlap information. Using the paired-end information to join the unique contigs into scaffolds, the single or multiple-scaffolds might represent individual chromosomes [52], [53]. For biologists, the most important data from the genome-sequencing projects could be the size and sequence files of chromosomes, scaffolds and whole genomes. Based on the information of these sequences, integrity and accuracy were calculated and used to evaluate the genome sequence quality.

The integrity of genome sequences was presented/evaluated by the ratio between chromosome size and genome size (CS/GS) (or between scaffold size and genome size (SS/GS)). This ratio of CS/GS (or SS/GS) may indicate the proportion of the spliced sequence to the whole genome sequence. A high ratio suggests a high level of genome sequence integrity, and many short reads being assembled into scaffold or chromosome sequences. On the other hand, a low CS/GS (or SS/GS) ratio indicates a low level of genome sequence integrity, and many short reads not being combined into scaffold or chromosome sequences.

In this study, we investigated 32 plant species (**Table 1**) whose entire genomes have been sequenced. Based on the released or unreleased chromosome sequences, these plants could be categorized into two groups: 1) chromosome sequence group (CSG) comprising of the species whose chromosome sequence data was available and 2) scaffold sequence group (SSG), consisting of species with only scaffold sequence data available. In the first group (CSG), the chromosome sequence data of 13 species have been released, with chromosome number ranging from 5 (*Arabidopsis thaliana* and *Brachypodium distachyon*) to 20 (*Glycine max*) and genome size ranging from 119 Mb (*Arabidopsis thaliana*) to 2,059 Mb (*Zea mays*). The ratio of CS/GS was such that *Malus* × *domestica* had the lowest value (70.89%) and *Brachypodium distachyon* the highest (99.63%) in the CSG plants. Among another 11 plant species, five (*Fragaria vesca*, *Solanum lycopersicum*, *Glycine max*, *Vitis vinifera* and *Zea mays*) had CS/GS ratios lower than 90%, ranging from 81.25% to 89.52%, while six species (*Sorghum bicolor*, *Populus trichocarpa*, *Oryza sativa*, *Medicago truncatula*, *Arabidopsis thaliana*, *Lotus japonicas*) exhibited ratios greater than 90%, with the range being between 90.27% and 98.09% (**Table 1**).

**Table 1:** The summary of plant genome sequencing projects.

	Scientific Name	Year	GenomeSize (Mb)	Chromosomeor ScaffoldSize (Mb)	(CS or SS)/GS %^a^	CS orSS No.^b^	Reference
Chromosome	*Arabidopsis thaliana*	2000	125	119	95.2	5	Nature, 408: 796–815
	*Brachypodium distachyon*	2010	272	271	99.63	5	Nature, 463: 763–768
	*Fragaria vesca*	2011	240	195	81.25	7	Nature Genetics, 43: 109–116
	*Glycine max*	2010	1,100	950	86.36	20	Nature, 463: 178–183
	*Lotus japonicus*	2008	472	463	98.09	6	DNA Research, 15(4): 227–239
	*Malus × domestica*	2010	742	526	70.89	17	Nature Genetics, 42: 833–839
	*Medicago truncatula*	2011	308	291	94.48	8	Nature, 480: 520–524
	*Oryza sativa*	2002	420	382	90.95	12	Science, 296(5565): 92–100
	*Populus trichocarpa*	2006	418	379	90.67	19	Science, 313(5793): 1596–1604
	*Solanum lycopersicum*	2012	900	760	84.44	12	Nature, 485: 635–641
	*Sorghum bicolor*	2009	730	659	90.27	10	Nature, 457: 551–556
	*Vitis vinifera*	2007	487	426	87.47	19	Nature, 449: 463–467
	*Zea mays*	2009	2,300	2,059	89.52	10	Science, 326(5956): 1112–1115
Scaffold	*Arabidopsis lyrata*	2011	207	207	100	695	Nature Genetics, 43: 476–481
	*Carica papaya*	2008	372	343	92.2	5,901	Nature, 452: 991–996
	*Citrus clementina*	–	–	296	–	1,128	www.citrusgenomedb.org
	*Citrus sinensis*	–	–	319	–	12,574	www.citrusgenomedb.org
	*Cucumis sativus*	2009	367	203	55.31	4,219	Nature Genetics, 41: 1275–1281
	*Eucalyptus grandis*	–	–	691	–	4,952	www.phytozome.net
	*Gossypium raimondii*	–	–	764	–	1,448	www.phytozome.net
	*Linum usitatissimum*	–	350	318	90.86	88,420	www.phytozome.net
	*Manihot esculenta*	–	760	533	70.13	12,977	www.phytozome.net
	*Mimulus guttatus*	–	430	322	74.88	2,216	www.phytozome.net
	*Panicum virgatum*	–	1,400	1,358	97	410,030	www.phytozome.net
	*Phaseolus vulgaris*	–	–	487	–	10,132	www.phytozome.net
	*Physcomitrella patens*	2008	–	480	–	2,106	Science, 319(5859): 64–69
	*Prunus persica*	–	230	227	98.7	202	www.phytozome.net
	*Ricinus communis*	2010	–	351	–	25,828	Nature Biotechnology, 28: 951–956
	*Selaginella moellendorffii*	2011	–	213	–	768	Science, 332(6032): 960–963
	*Setaria italica*	–	515	401	77.86	336	www.phytozome.net
	*Solanum tuberosum*	2011	840	734	87.38	68,169	Nature, 475: 189–195
	*Thellungiella halophila*	–	–	243	–	639	www.phytozome.net

a, the percentage of chromosome or scaffold size in genome size.

b, the number of chromosome or scaffold sequences.

–, means unknown.

In the second group (SSG), only 19 plant species have the scaffold sequence released. In this group, *Arabidopsis lyrata* had the highest ratio of SS/GS being close to 100%, for its scaffold size and genome sizes were both 207 Mb. The ratio of SS/GS in *Cucumis sativus* was the lowest (55.31%) in the SSG plants, because of the significant difference between scaffold size (203 Mb) and genome size (367 Mb). *Panicum virgatum* had the highest scaffold size (1,358 Mb) and genome size (1,400 Mb) in the SSG plants, and the ratio was 97%. In addition to the nine plant species whose genome size information had not been released, the other seven species (*Manihot esculenta*, *Mimulus guttatus*, *Setaria italica*, *Solanum tuberosum*, *Linum usitatissimum*, *Carica papaya* and *Prunus persica*), have SS/GS ratios of, 70.13%, 74.88%, 77.86%, 87.38%, 90.86%, 92.20% or 98.70% respectively.

The generation of chromosome sequences ideally passes through four stages: the stage of from Genomic DNA to short sequencing reads, short sequencing reads to contig sequences, contig sequences to scaffold sequences, and from scaffold sequences to chromosome sequences [52]. From the above analysis, it can be explained that the ratio of CS/GS (SS/GS) in most plants studied were lower than 90%, and a lot of short reads or contig sequences had not been assembled into scaffold sequences or some scaffold sequences had not been assembled into chromosome sequences.

### EST Coverage and Distribution

EST was considered to be a piece of the corresponding expressed genes located in the genome, and it was physically mapped to individual chromosomes or chromosome intervals for the chromosome bin map construction [54]. When genomic DNA was used to construct sequencing libraries and then sequenced, short reads were generated from both ends of the clones, and finally the short reads were assembled into scaffold or chromosome sequences [23], [52]. Considering the character of ESTs, genes and genomes as well as their relationships, EST could be mapped into the scaffold or chromosome sequences, and the ratio between matched ESTs and selected ESTs (mEST/sEST) could be used in the evaluation of accuracy of genome sequences. A high accuracy of genome sequences could be proven by high ratios of mEST/sEST.

In this study, 181,786 randomly selected clean EST sequences and 15,970 Mb chromosome (scaffold) sequences were downloaded and/or generated then subjected to analysis of mEST/sEST ratios in 32 plants (**Table 2**). Based on the number of ESTs in dbEST, nineteen SSG plants could be divided into three groups with the first consisting of *Arabidopsis lyrata* and *Cucumis sativus* both having lower than 10,000 ESTs in dbEST. After preprocessing, all the clean ESTs (548 and 7,396 respectively) were aligned with scaffold sequences for mEST/sEST analysis. From the clean ESTs, 95.44% (523) and 95.42% (7,057) were matched on the scaffold sequences in *Arabidopsis lyrata* and *Cucumis sativus*, respectively. Ten SSG plant species (*Carica papaya*, *Eucalyptus grandis*, *Gossypium raimondii*, *Manihot esculenta*, *Physcomitrella patens*, *Prunus persica*, *Ricinus communis*, *Selaginella moellendorffii*, *Setaria italica* and *Thellungiella halophile*) with a total of 10,000 to 100,000 ESTs each were clustered into the second group. The ratios of mEST/sEST in this group were 89.86%, 94.03%, 92.40%, 94.05%, 95.80%, 95.01%, 96.21%, 98.85%, 92.26% and 98.34%, respectively. The remaining seven SSG plants were placed in the third group where each species had more than 100,000 ESTs and their mEST/sEST ratios were 91.77%, 89.02%, 98.54%, 96.19%, 97.18%, 92.58% and 97.72% for *Citrus clementina*, *Citrus sinensis*, *Linum usitatissimum*, *Mimulus guttatus*, *Panicum virgatum*, *Phaseolus vulgaris* and *Solanum tuberosum*, respectively. From the above mEST/sEST ratios in SSG plants, it is discernible that a small percentage (1.15%–10.98%) of ESTs could not be mapped on the scaffold sequences, and it is indicative that there was a proportion of mismatches and losses during assembly, and thus the generated scaffold sequences in some cases was not satisfactory. Furthermore some valuable gene information may exist in the “junk” sequences, produced from the unassembled short reads.

**Table 2:** The alignment results of EST sequences in thirty-two plant species.

	Plant species	EST numberin dbEST	EST numberin alignment	EST %^a^	Matched ESTnumber	mEST/sEST %^b^
Chromosome	*Arabidopsis thaliana*	1,529,700	15,297	1.00	14,020	91.65
	*Brachypodium distachyon*	128,092	1,281	1.00	1,259	98.28
	*Fragaria vesca*	10,855	1,086	10.00	958	88.21
	*Glycine max*	1,461,624	14,616	1.00	14,207	97.20
	*Lotus japonicus*	242,432	2,424	1.00	1,283	52.93
	*Malus* × *domestica*	324,847	3,248	1.00	2,957	91.04
	*Medicago truncatula*	269,238	2,692	1.00	1,874	69.61
	*Oryza sativa*	1,252,989	12,530	1.00	12,016	95.90
	*Populus trichocarpa*	89,943	8,994	10.00	8,726	97.02
	*Solanum lycopersicum*	298,306	2,983	1.00	2,907	97.45
	*Sorghum bicolor*	209,835	2,098	1.00	2,017	96.14
	*Vitis vinifera*	446,639	4,466	1.00	4,099	91.78
	*Zea mays*	2,019,137	20,191	1.00	19,082	94.51
	Total/Average	8,283,637	91,906	2.38	85,405	89.36
Scaffold	*Arabidopsis lyrata*	561	548	100.00	523	95.44
	*Carica papaya*	77,393	7,739	10.00	6,954	89.86
	*Citrus clementina*	117,865	1,179	1.00	1,082	91.77
	*Citrus sinensis*	214,142	2,141	1.00	1,906	89.02
	*Cucumis sativus*	8,146	7,396	100.00	7,057	95.42
	*Eucalyptus grandis*	42,576	4,258	10.00	4,004	94.03
	*Gossypium raimondii*	63,577	6,358	10.00	5,875	92.40
	*Linum usitatissimum*	286,852	2,869	1.00	2,827	98.54
	*Manihot esculenta*	80,631	8,063	10.00	7,583	94.05
	*Mimulus guttatus*	231,095	2,311	1.00	2,223	96.19
	*Panicum virgatum*	720,590	7,206	1.00	7,003	97.18
	*Phaseolus vulgaris*	123,988	1,240	1.00	1,148	92.58
	*Physcomitrella patens*	20,456	2,046	10.00	1,960	95.80
	*Prunus persica*	79,815	7,982	10.00	7,584	95.01
	*Ricinus communis*	62,592	6,259	10.00	6,022	96.21
	*Selaginella moellendorffii*	93,811	9,381	10.00	9,273	98.85
	*Setaria italica*	66,027	6,603	10.00	6,092	92.26
	*Solanum tuberosum*	249,920	2,499	1.00	2,442	97.72
	*Thellungiella halophila*	38,022	3,802	10.00	3,739	98.34
	Total/Average	2,578,059	89,880	16.16	85,297	94.77

a, the percentage of randomly selected clean EST sequences in total EST sequences.

b, the percentage of matched EST sequences in randomly selected clean EST sequences.

Except for *Lotus japonicus* (52.93%) and *Medicago truncatula* (69.61%), the ratios of mEST/sEST ranged between 88.21% and 98.28% in the other CSG plants, with the specific scores being 88.21% (*Fragaria vesca*), 91.04% (*Malus* × *domestica*), 91.65% (*Arabidopsis thaliana*), 91.78% (*Vitis vinifera*), 94.51% (*Zea mays*), 95.90% (*Oryza sativa*), 96.14% (*Sorghum bicolor*), 97.02% (*Populus trichocarpa*), 97.20% (*Glycine max*), 97.45% (*Solanum lycopersicum*) and 98.28% (*Brachypodium distachyon*). The ratio between unmatched ESTs and randomly selected clean ESTs ranged from 1.72% to 47.07% in the CSG plants indicating that some sections of the ESTs could not mapped on the chromosome sequences. It was also found that the average ratio of mEST/sEST in the SSG plants (94.77%) was higher than in the CSG plants (89.36%). These results also indicate that many scaffold sequences or other informative sequences were lost during assembly, and the accuracy of genome sequences was as high as envisaged. In some cases where the chromosome sequence had been released it was found that despite a high integrity of chromosome sequence, its accuracy was lower than predicted thus creating an element of doubt on the reliability of the genome sequence. Examples of such instances include *Lotus japonicus* and *Medicago truncatula*.

**Figure 1:**
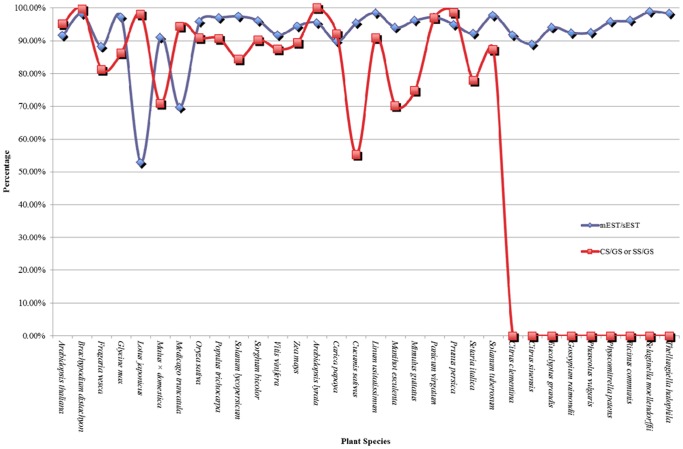
Ratios of mEST/sEST and CS/GS (SS/GS) in thirty-two plants. The blue line shows the ratios of mEST/sEST and the red line shows the ratios of CS/GS (SS/GS) in thirty-two plants. Because genome size information of nine plants was minssing, the ratios of SS/GS were 0%.

Because of the lack of genome size information in nine of the 32 plant species selected for this study, we only compared the ratios of CS/GS (SS/GS) and mEST/sEST in 23 plants (**Figure 1**). Comparison of the two ratios reveals a positive relationship (when the ratio of CS/GS (SS/GS) was increased at that time the ratio of mEST/sEST was also increased, and vice versa) in all the 23 plant species apart from *Lotus japonicus*, *Medicago truncatula* and *Malus* × *domestica*. The ratios of mEST/sEST in SSG plants averaged 90%, with a range of 92.26% to 96.19% in *Cucumis sativus*, *Manihot esculenta*, *Mimulus guttatus* and *Setaria italica* whose SS/GS ratios were 55.31%, 70.13%, 74.88% and 77.86% respectively. The high mEST/sEST ratio and low SS/GS ratio in the plants showed both a low scaffold sequence integrity and a high scaffold sequence accuracy with most genes being found in the scaffold sequence (**Table 1** and **Table** **2**). In the CSG plants, *Lotus japonicus*, *Medicago truncatula* and *Malus* × *domestica* differed from the other species when the ratios of mEST/sEST and CS/GS were compared. Although the ratios of CS/GS were high (98.09% and 94.48%) in *Lotus japonicus* and *Medicago truncatula*, and their mEST/sEST ratios were low (52.93% and 69.61%), an indication that quite a number of important genes are located in the 1.91% or 5.52% of the sequences, that have not been assembled into the chromosomes and that the accuracy of chromosome sequences was low. For *Malus* × *domestica*, the ratio of CS/GS was low (70.89%), but the mEST/sEST ratio was high (91.04%), a pointer of low integrity and high accuracy in the apple chromosome sequence. A few genes were therefore contained in the unassembled scaffold sequence. The CS/GS ratio could be above 90% in the further published version of the apple chromosome.

With only thirteen plants having their chromosome sequences released publicly, we investigated the matched EST and matched frequencies in every 0.1 Mb (100,000 bp) segment of the CSG plant species (**Table S1**). Among the 15,297 clean randomly selected *Arabidopsis thaliana* EST sequences, 14,020 (91.65%) could be mapped on the chromosomes, with the average proportion of segments with matching ESTs being 92.03%. In *Zea mays*, the average proportion was 97.38%, which was the highest average in these 13 plants. In *Vitis vinifera*, the average proportion was 40.34%, which was lower than *Arabidopsis thaliana and Zea mays.* The percentages of segments with matching ESTs in the other 10 plants are as shown in **Table S1**. Finally, the CSG plants could be categorized into three groups according to the average percentage of segments with matching ESTs in each plant. *Zea mays* (97.38%), *Arabidopsis thaliana* (93.40%), and *Oryza sativa* (72.82%) were in the first group having average percentage of segments with matching ESTs being larger than 70%. *Populus trichocarpa* (average 51.18%), *Glycine max* (41.13%), *Vitis vinifera* (40.34%), *Medicago truncatula* (37.29%), *Brachypodium distachyon* (33.49%), *Malus* × *domestica* (33.40%), *Fragaria vesca* (31.06%) were the second group whose average percentage of segments with matching ESTs ranging from 30% to 55%. The percentages of segments with matching ESTs in the last group (*Solanum lycopersicum* (20.83%), *Sorghum bicolor* (18.31%) and *Lotus japonicas* (12.88%)) ranged from 10% to 25%. High ratios of mEST/sEST and high percentages of segments with matching ESTs are characteristics of high quality chromosome sequences in plant species. From the above analysis in the CSG plants, only plant species in the first group have high percentages of segments with matching ESTs (larger than 70%), while in the other ten plants these percentage was low. Combining information of the percentage of segments with matching ESTs and the ratio of mEST/sEST, we can infer that many ESTs were mismatched on the chromosome sequences, many segments in the chromosome were still unmatched, and a number of chromosome sequences were incomplete sequences. Even though some chromosome sequences had a high ratio of CS/GS, the accuracy of chromosome sequences were still lower than previously thought.

EST-Chromosome mapping analysis comprises of two aspects, one being the percentage of segments with matching ESTs while the other is the number of ESTs matched in each segment. Due to ESTs being partially expressed genes in chromosomes, we can identify the distribution density of genes in the chromosome sequence of CSG plants using the number of EST matches in each segment.

**Figure 2:**
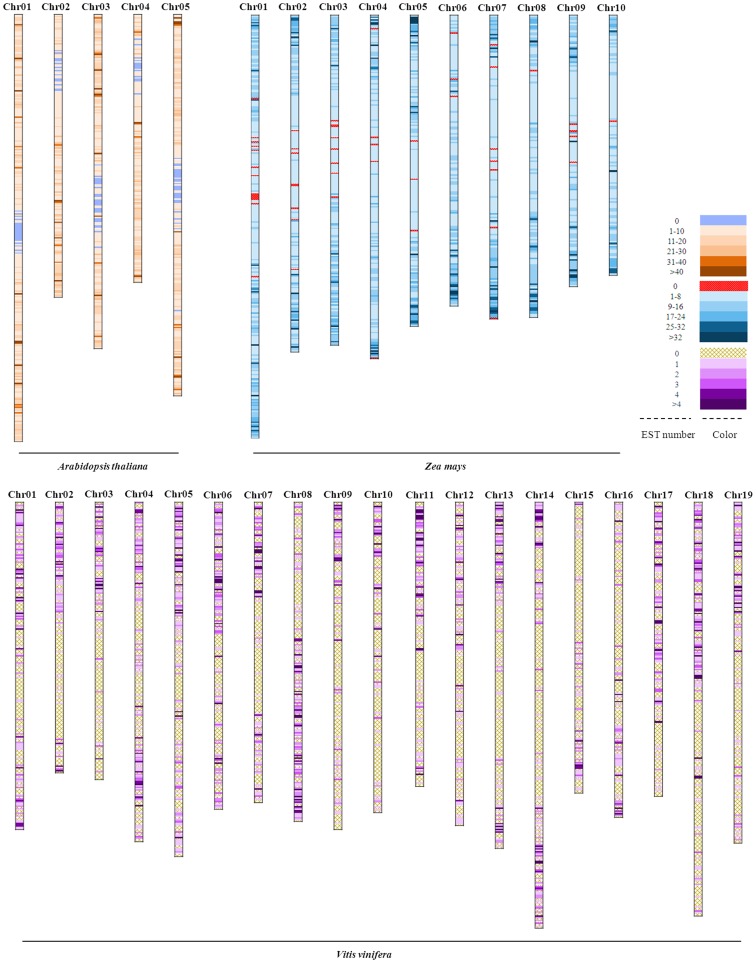
Distribution map of EST coverage and abundance of *Arabidopsis thaliana*, *Zea mays* and *Vitis vinifera*. The species name is shown at the end of each chromosome model, and different gradient colors describe the abundance in each plant. Based on the alignment results, the abundance of EST sequences were divided into six parts, including the EST matched times of 0, 1–10, 11–20, 21–30, 31–40, >40 in *Arabidopsis thaliana*; 0, 1–8, 9–16, 17–24, 25–32, >32 in *Zea mays*; 0, 1, 2, 3, 4, >4 in *Vitis vinifera*, respectively.

Based on the chromosome numbers and importance in research, *Arabidopsis thaliana* (5 chromosomes), *Zea mays* (10 chromosomes), and *Vitis vinifera* (19 chromosomes) were used as the examples in the detailed analysis of EST matching frequency in each segment and a draft sketch of EST matched segments and frequency was as shown in **Figure 2**. Draft sketches of the other ten CSG plants were also analyzed and presented in **Figure S1**. From the analysis of *Arabidopsis thaliana*, *Zea mays* and *Vitis vinifera*, it is discernible that though most EST sequences were mapped at both ends of chromosomes, a few were found on the middle parts. In *Arabidopsis thaliana*, the non-EST regions were found at the middle parts of chromosomes 1, 3 and 5, and near the middle part of chromosomes 2 and 4. In *Zea mays*, these non-ESTs regions were found at the middle part of each chromosome except chromosomes 6 and 8. In *Vitis vinifera*, mass non-ESTs regions were found as shown in the draft sketch (**Figure 2**), suggesting that the quality of chromosome sequences in *Vitis vinifera* was lower than in *Arabidopsis thaliana* and *Zea mays*. Generally, the genome-EST non-matched regions in these three plants were mainly located at the middle regions of the chromosome, and fewer were located at the near middle region of the chromosome. The other ten CSG plants reflected a similar situation (**Figure S1)**.

EST matched times can be used as an important index to evaluate the quality of chromosome sequence, which can be proved by the high matched times of EST-Chromosome, and also important in comparative analysis of chromosome structure and evolution, targeted genome sequencing for a large genome species [34]. The high ESTs matched times in the CSG plants (**Figure 2** and **Figure S1)** were distributed at the two ends of chromosome, the gene density was high, which was displayed as deep color. Along with the remove from the end of chromosome to the middle part of chromosome, the ESTs matched time was decreased, and the gene density was low, which was displayed as light color.

### GC Content in CSG

**Figure 3:**
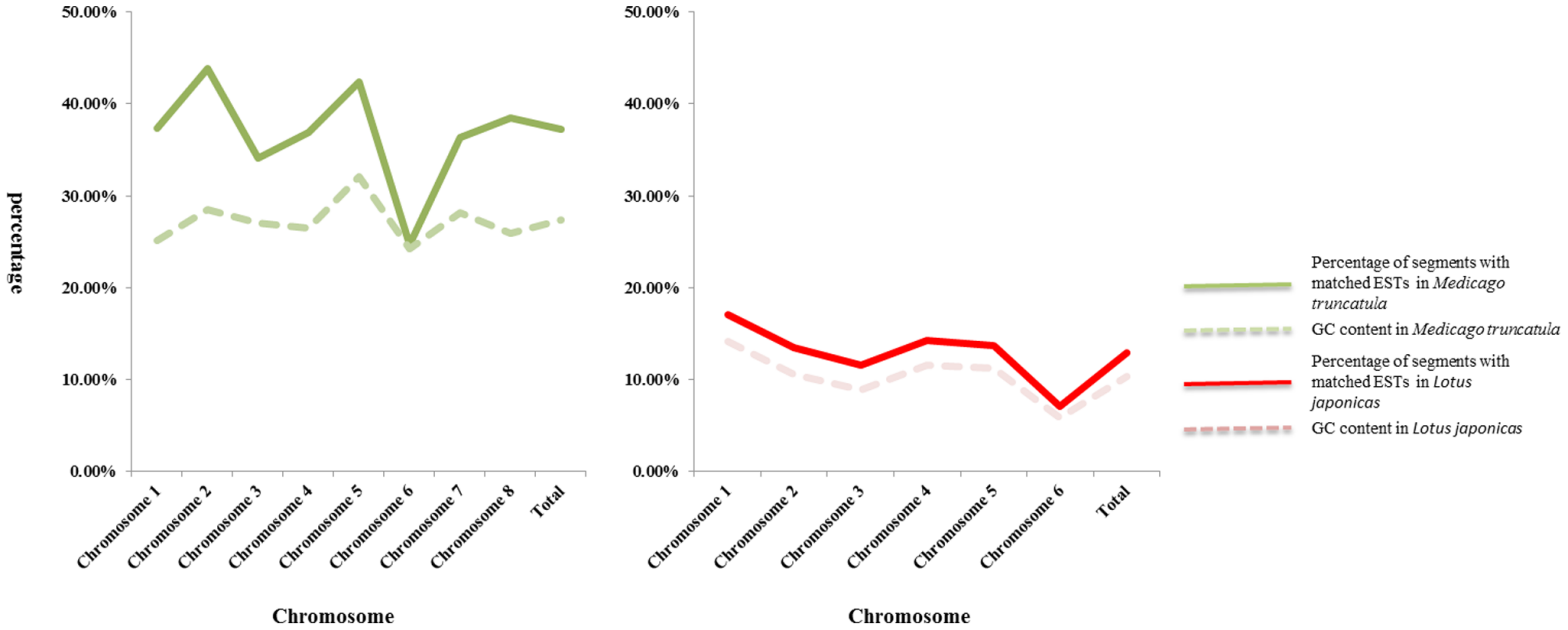
Distribution map of the percentage of segments with matching ESTs and the GC content in *Medicago truncatula* and *Lotus japonicas*. The green solid line represents the percentage of segments with matching ESTs in *Medicago truncatula*, and the green dotted line represents the GC content in *Medicago truncatula*. The red solid line represents the percentage of segments with matching ESTs in *Lotus japonicas*, and the red dotted line represents the GC content in *Lotus japonicas*.

Both GC-poor and GC-rich sequences will affect the quality of genome sequence (chromosome or scaffold sequence) by increasing the sequencing errors and assemble difficulty [55], [56]. The GC content difference is also a primary factor for non-random sequencing-depth distribution [57]. In this study, the GC contents of 13 CSG plants were analyzed with GCcontent.pl script. The average GC content in CSG plants were 35.97% (*Arabidopsis thaliana*), 46.21% (*Brachypodium distachyon*), 35.78% (*Fragaria vesca*), 34.21% (*Glycine max*), 10.34% (*Lotus japonicas*), 27.62% (*Malus* × *domestica*), 27.39% (*Medicago truncatula*), 42.41% (*Oryza sativa*), 32.84% (*Populus trichocarpa*), 32.11% (*Solanum lycopersicum*), 41.56% (*Sorghum bicolor*), 33.67% (*Vitis vinifera*) and 46.60% (*Zea mays*) (**Table S2**). Based on the average GC content, 13 plants were divided into three groups. The first group was *Brachypodium distachyon*, *Oryza sativa*, *Sorghum bicolor* and *Zea mays*, the average GC content was larger than 40%. The second group was *Lotus japonicas*, *Malus* × *domestica* and *Medicago truncatula*, the average GC content was lower than 30%. The rest of plants were belonged to the third group and the average GC content was between 32.11% to 35.97%. There were significantly GC content differences among 13 CSG plants. It was noticed that there were no significant GC content difference among the chromosomes in most CSG plants. In *Arabidopsis thaliana*, it was ranged from 35.68% to 36.32%, and in *Vitis vinifera*, the value of GC content was ranged from 32.86% to 34.09%. However, the GC content in *Lotus japonicas* was varied from 5.77% to 14.07%, with 8.3 percentage points difference, and in *Medicago truncatula*, the value of GC content was varied from 24.25% to 32.03%, with 7.78 percentage points difference. The ratios of mEST/sEST and the percentages of segments with matching ESTs of *Lotus japonicas* and *Medicago truncatula* were lowest in the CSG plants. Comparison with the GC content and the percentages of segments with matching ESTs in every chromosome of *Lotus japonicas* and *Medicago truncatula* (**Figure 3**), the positive correlation relationship was found. The value of GC content was increased while the percentage of segments with matching ESTs was raise, and vise verse. This relationship in *Lotus japonicas* was particularly prominent.

Comparison of the average GC content with the ratio of CS/GS and the ratio of mEST/sEST (**Table 3**) were processed in the CSG plants. The result indicated that the ratio of CS/GS and the ratio of mEST/sEST were higher than 80% in the CSG plants with the average GC content was between 30%–47%. When the average GC content was raised to 46%, the ratio of CS/GS and the ratio of mEST/sEST were still higher than 88%. But when the average GC content was lower than 30%, especially the *Lotus japonicas*, the average GC content was only 10.34%, the ratio of CS/GS and the ratio of mEST/sEST showed a significantly decrease trend. The comparison showed that in some plant, the integrity of genome sequence was high, but the accuracy of genome sequence was low, such as *Lotus japonicas* and *Medicago truncatula*. In some plant, the accuracy of genome sequence was high, but the integrity of genome sequence was low, such as *Malus* × *domestica*. The result indicated that when the GC content was between 30%–47%, it will not act as a major affecting factor in genome sequence quality, and it may tolerated by the Genome sequencer and assembler. When the average GC content was lower than 30%, it will cause the significantly affect in genome sequence quality, and the influence on accuracy larger than integrity. The GC content proportions in the CSG plants were content with the previous reports in mammalian [57], [58], the moderate GC content will not improve the difficult of genome sequencing, and not affect the genome short reads assemble, gene prediction from the genome sequences.

**Table 3:** The comparison among the values of average GC content, CS/GS and mEST/sEST in CSG plants.

Plant species	Average GC content	CS/GS proportion	EST matching proportion
*Arabidopsis thaliana*	35.97%	95.20%	91.65%
*Brachypodium distachyon*	46.21%	99.63%	98.28%
*Fragaria vesca*	35.78%	81.25%	88.21%
*Glycine max*	34.21%	86.36%	97.20%
*Lotus japonicus*	10.34%	98.09%	52.93%
*Malus × domestica*	27.62%	70.89%	91.04%
*Medicago truncatula*	27.39%	94.48%	69.61%
*Oryza sativa*	42.41%	90.95%	95.90%
*Populus trichocarpa*	32.84%	90.67%	97.02%
*Solanum lycopersicum*	32.11%	84.44%	97.45%
*Sorghum bicolor*	41.56%	90.27%	96.14%
*Vitis vinifera*	33.67%	87.47%	91.78%
*Zea mays*	46.60%	89.52%	94.51%

### Repeat DNA Sequence Distribution and its Relationship with Matched ESTs Distribution in CSG Plants

Repeat DNA sequence was the major problem in the *de novo* sequencing assemble, it can usually affect the assemble quality of genome sequences [58]. The interspersed repeat sequence, low complexity sequence and simple repeat sequence were identified by RepeatMasker software. In this study, we only discuss these three type of repeat sequences. To investigate the relationship between repeat DNA sequence and genome sequence quality, we did the EST-Chromosome mapping using the whole EST sequences stored in dbEST and identify the repeat DNA in chromosomes, among which *Arabidopsis thaliana* (Chromosome No.  = 5), *Brachypodium distachyon* (Chromosome No.  = 5), *Glycine max* (Chromosome No.  = 20), *Oryza sativa* (Chromosome No.  = 12), *Vitis vinifera* (Chromosome No.  = 19), and *Zea mays* (Chromosome No.  = 10) were analyzed in detail considering their research and economical importance. All the clean ESTs were used as query for EST mapping to chromosome sequences and thirty segments of three chromosomes with 0.1 Mb (100,000 bp) in length were randomly selected for this analysis.

**Figure 4:**
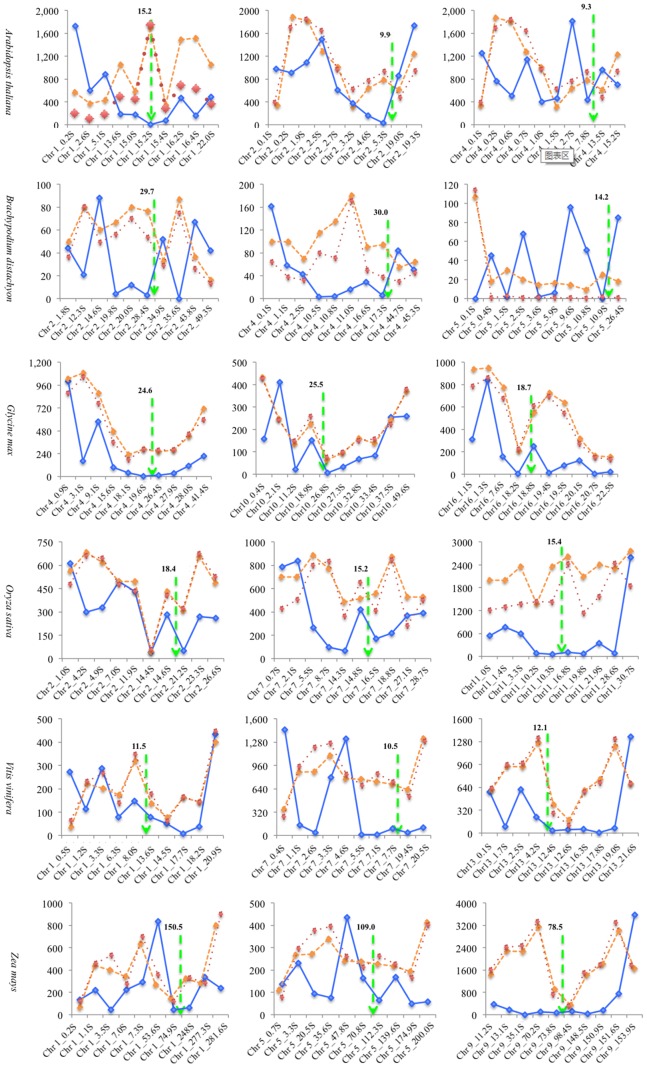
Distribution map of EST and repeat sequence abundance, and length in ten chromosome regions of *Arabidopsis thaliana*, *Brachypodium distachyon*, *Glycine max*, *Oryza sativa*, *Vitis vinifera*, and *Zea mays*. The blue line represents the changing trend in EST abundance, the yellow line represents repeat sequence abundance, the red line represents repeat sequence length, and the green line indicates the middle point of chromosomes. The value of Y-axis indicates the number of EST abundance.

As mentioned above that the EST distribution analysis (**Figure 2**) showed most randomly selected clean EST sequences in the CSG plants were located at the both ends of each chromosome, and fewer were located in the middle region of the chromosome. The analysis of segments (S, the length of each S was 0.1 Mb) also indicated the similar result that much ESTs were matched in the region near ends site of chromosome, and few ESTs were matched with the region near in the middle site of chromosome. After EST-Chromosome mapping analysis, a draft sketch was drawn, in which the blue solid line was used to show the changing trend of matched EST number in each selected ten regions, and the green dashed line was used to indicate middle point of the chromosome (**Figure** **4**, **Table S3**).

The EST matched times in the segments near the two ends of chromosome was larger than the one in the segments near the middle site of chromosome. For example, in *Arabidopsis thaliana*, ten segments were randomly selected from chromosome 1, including 0.2 S region (200,000 bp–300,000 bp), 2.6 S region (2,600,000 bp–2,700,000 bp), 5.1 S region (5,100,000 bp–5,200,000 bp), 13.6 S region (13,600,000 bp–13,700,000 bp), 15.0 S region (15,000,000 bp–15,100,000 bp), 15.2 S region (15,200,000 bp–15,300,000 bp), 15.4 S region (15,400,000 bp–15,500,000 bp), 16.2 S region (16,200,000 bp–16,300,000 bp), 16.4 S region (16,400,000 bp–16,500,000 bp), and 22.0 S region (22,000,000 bp–22,100,000 bp). The ESTs matched times of 15.2 S region (the green line indicated the middle point of chromosome) was only 2 ESTs, it was the lowest times of EST matched in the ten segments of *Arabidopsis thaliana* Chromosome 1 (**Table S3**). The changing trend of EST matched times was increased when the selected segments were near the ends site of chromosome, the ESTs matched times in the 15.0 S region to the 0.2 S region ranged from 172 to 1,726, and in the 15.4 S region to the 22.0 S region, the ESTs match times ranged from 69 to 483. From 0 S region to 11.5 S region on grape chromosome 1, five segments were randomly selected, the segments and the EST matched times were 0.5 S (272), 1.2 S (114), 3.3 S (287), 6.3 S (78) and 8.0 S (147). From 11.5 S region to 23.0 S region on grape chromosome 1, other five segments were also randomly selected, the segments and the EST matched times were 13.6 S (78), 14.5 S (51), 17.7 S (8), 18.2 S (38) and 20.9 S (433). The EST matched times in these segments showed the characteristics that it will decrease from the two ends of the chromosome to the middle site of the chromosome. This changing trend was also observed in the other plants, such as *Brachypodium distachyon, Glycine max, Zea mays* and *Oryza sativa*. The distribution law of EST matched times in segments was corresponding with that in whole chromosomes.

Three types of repeat sequence were identified by RepeatMasker software. The changing trends both of repeat DNA sequence times and total length in the 0.1 Mb randomly selected segment was basically opposite to the distribution law of EST matched times in the 0.1 Mb randomly selected segment. In the two ends of the chromosome, the repeat sequence times and total lengths were high (long). In the middle site of the chromosome, the repeat sequence times and total lengths were low (short). This distribution law in some chromosomes was particularly evident. In *Arabidopsis thaliana*, the peak point region (15.2 Mb region) of the ten randomly selected 0.1 Mb segments on chromosome 1 got the highest value of repeat sequence matched times and total length (84/12,258 bp), but in the segments near the two ends site of chromosome 1, the ebb point segments only got the low repeat sequence matched times and total length value of 18/735 bp in 2.6 S segment and 52/2,508 bp in 22.0 S region. The same distribution law was found in *Oryza sativa* chromosome 4.

Sometimes, the distribution law of repeat sequence times and total lengths in the 0.1 S region was the same with the distribution law of EST matched times in the 0.1 S region. In *Glycine max*, the value of repeat sequence times and total length in the segment near the ends site of chromosome were higher than they in the near of the middle site of chromosome. For example, in *Glycine max* chromosome 4, the repeat sequence times and total length in 0.9 S region, 3.1 S region and 41.4 S region were 171/10,118 bp, 181/12,021 bp, and 119/6,877, respectively, but the repeat sequence times and total length in 19.6 S region, which were near the middle site of chromosome, was only 48/3,346 bp. The same distribution law was found in *Vitis vinifera* chromosome 13.

**Figure 5:**
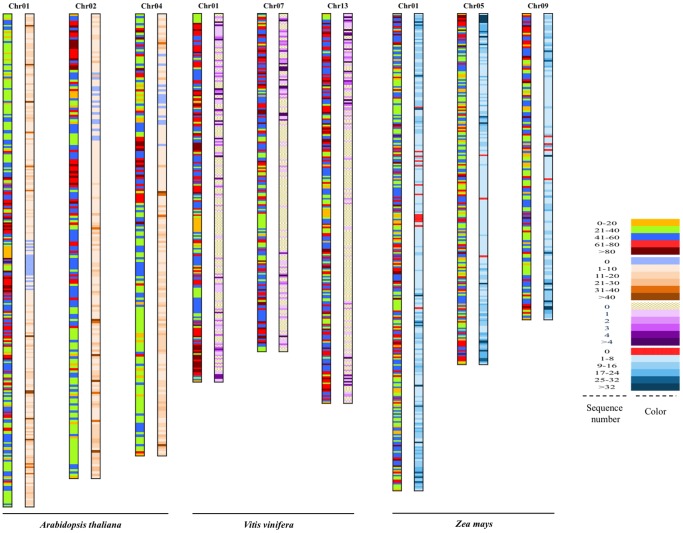
Distribution sketch of ESTs and repeat sequences in three randomly selected chromosomes from *Arabidopsis thaliana*, *Vitis vinifera* and *Zea mays*. The sketch on the left is the distribution of repeat sequences in chromosome; the right one indicates EST sequence distribution on the same chromosome.

The relationship of EST matched times and repeat sequence matched times in the whole chromosome was also analyzed, and the situations in *Arabidopsis thaliana*, *Vitis vinifera* and *Zea mays* were used (**Figure 5**) as examples. From the whole chromosome analysis, there were two major kinds of distribution law of repeat sequence, where relatively higher distribution was in the regions near the middle part of chromosome, and lower distribution was found in the regions near two ends of the chromosome, such as *Arabidopsis thaliana* chromosome 1 and 4; another one was relatively higher distribution in the regions near the ends of chromosome and lower in the middle part of chromosome, such as *Vitis vinifera* chromosome 7 and 13.

Comparison of the distribution of repeat sequence and EST sequence on chromosomes, EST sequence and repeat sequence showed some different modes. One was the EST matched times in the regions with high repeat sequence density was lower than it in the regions with low repeat sequence density, such as *Arabidopsis thaliana* chromosome 1 and *Zea mays* chromosome 1. Another was on the contrary, where the EST matched times in the region with high repeat sequence density was higher than it in the regions with low repeat sequence density, such as *Vitis vinifera* chromosome 1, 7 and 13. At the same time, we also found that the regions with less EST matched times were between two regions with high repeat sequence density in the chromosome of some plants, such as the middle region of *Arabidopsis thaliana* chromosome 1 and the end part of *Arabidopsis thaliana* chromosome 2.

Tandemly repeated DNA was the other kind of repeat sequence, except for the interspersed repetitive DNA, which was identified by RepeatMasker [59], [60]. Our analysis indicated that the repeat sequence will affect the distribution of genes on the chromosome, and also the important affect factor of genome sequence quality, it will raise the difficult of short reads assemble.

From the above analysis (**Table 1**), the average ratio of SS/GS (97%) were larger than the average ratio of CS/GS (84.44%), the result indicated that a large number of sequence were not assembled into chromosome sequence, and they were taken as “Junk sequence” or “Random sequence” [61]. The ratio of mEST/sEST in the SSG plants was 94.77%, which was larger than the CSG plants (89.36%). From the EST-Chromosome mapping analysis, most matched ESTs were found in the two end regions of chromosome, and few matched ESTs were concentrated at the middle site of chromosome. According to the above analysis, thirteen CSG plants were divided into four groups. *Arabidopsis thaliana*, *Oryza sativa* and *Zea mays* belonged to the first group (CS/GS proportion and EST matching proportion >85%, the proportion of segments with matching EST >70%, Sanger sequencing platform), and *Brachypodium distachyon*, *Populus trichocarpa*, *Vitis vinifera* and *Glycine max* belonged to the second group (CS/GS proportion and EST matching proportion >85%, the proportion of segments with matching EST >30%,). The third group contained *Sorghum bicolor*, *Solanum lycopersicum* and *Fragaria vesca* (CS/GS proportion and EST matching proportion >80%), while *Lotus japonicus*, *Medicago truncatula* and *Malus* × *domestica* were belonging to the last group (CS/GS proportion or EST matching proportion <80%). Assemble the scaffold sequence into chromosome sequence should be the primary task for the other nineteen plants. Low GC content and repeat DNA has the influence on the genome sequence assemble.

### The Influence of Low Quality Genome Sequence on the Genomics Downstream Analysis

**Figure 6:**
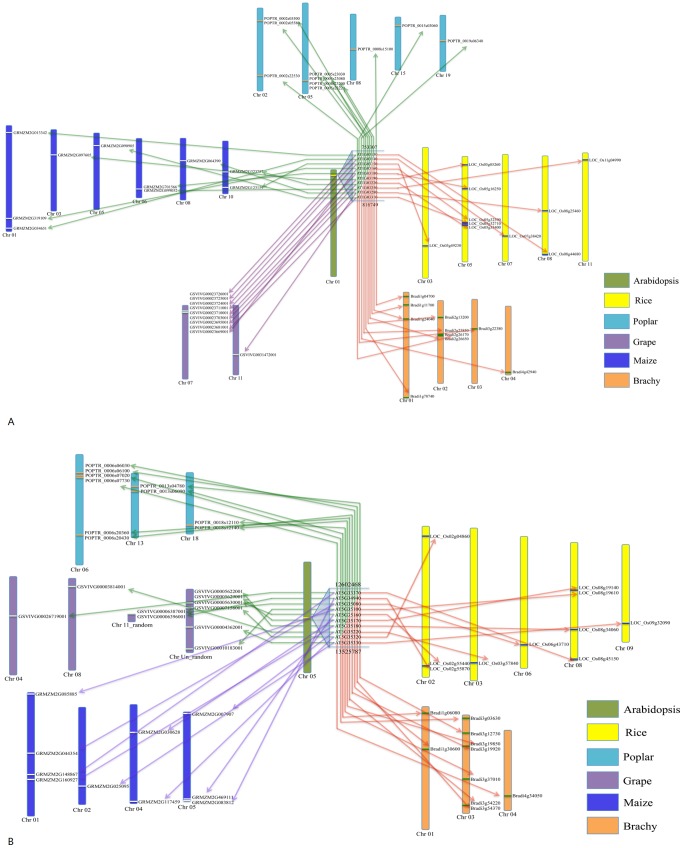
Draft distribution of gene cluster 1 (A) and 9 (B) in *Arabidopsis thaliana*, *Brachypodium distachyon*, *Zea mays*, *Oryza sativa*, *Populus trichocarpa* and *Vitis vinifera*.

Genome sequences were assemblies form the basis of genome research. Any errors could directly impair genomic and comparative genomic predictions and inferences based upon them [62]. For the collinearity analysis in *Arabidopsis thaliana*, *Brachypodium distachyon*, *Oryza sativa*, *Populus trichocarpa*, *Vitis vinifera*, and *Zea mays*, we identified ten adjacent gene clusters (100 genes in total in five chromosomes) in *Arabidopsis thaliana* at first, and then obtained the homologue genes in the other plants. The location of homologue genes on chromosome was retrieved from the genome annotation of each plant (**Table S4**), and the collinearity sketch was drawn (**Figure 6** **,** **Figure S2**).

Gene cluster 1 and 9 were taken as the example to describe the influence of low quality genome sequence on the collinearity analysis in six plants (**Figure 6**). In gene cluster 1 (**Figure 6A**), nine genes were distributed in the adjacent regions on *Vitis vinifera* chromosome 7, and eight genes (80%) were shown the collinearity relationship with *Arabidopsis thaliana* homologous genes (GSVIVG00023726001-AT1G03130, GSVIVG00023725001-AT1G03140, GSVIVG00023724001-AT1G03150, GSVIVG00023711001-AT1G03180, GSVIVG00023710001-AT1G03190, GSVIVG00023703001-AT1G03220, GSVIVG00023695001-AT1G03250 and GSVIVG00023681001-AT1G03310). In other three plants, only 40%, 30% and 20% gene were shown the collinearity relationship with *Arabidopsis thaliana* homologous genes. The collinearity levels existed in *Brachypodium distachyon*, *Oryza sativa* and *Zea mays* were lower than *Vitis vinifera*.

Once the quality of genome sequence is low, the collinearity analysis will be affected. In gene cluster 9 (**Figure 6B**), two genes of *Brachypodium distachyon*, *Oryza sativa* and *Zea mays* were shown the collinearity relationship with *Arabidopsis thaliana*. Eight homologous genes of grape were located on the unassembled sequence, namely: two gene were located on the chromosome 11_random sequence (a supercontig sequence, only know it belongs to chromosome 11, but the information about location on chromosome 11 is unknown), six homologous genes were located on the chromosome unknown_random sequence (a supercontig sequence with no information about the chromosome and location). Three homologous genes located on chromosome unknown_random sequence (GSVIVG00005630001-AT5G35160, GSVIVG00005629001-AT5G35170 and GSVIVG00005622001-AT5G35180) were shown the collinearity relationship with *Arabidopsis thaliana*. Because of lack of true information about chromosome and location of these three genes, the collinearity analysis in these three genes should be doubted. The similar results obtained in gene cluster 3 (two grape gene were located on chromosome 1_random and chromosome 15_random sequence, respectively), gene cluster 5 (two grape genes were located on chromosome unknown_random sequence), gene cluster 6 (one grape gene was located on chromosome unknown_random sequence), gene cluster 7 (one grape gene was located on chromosome 18_random sequence and two grape genes were located on chromosome unknown_random sequence) and gene cluster 10 (two grape genes were located on chromosome unknown_random sequence). The low quality of genome sequence increase collinearity analysis difficulty among plants. The bioinformatics tools and algorithm should be improved in the current scientific era, especially many plants genome sequence were finished and being publicly in a dramatic speed. The high quality of genome sequences will be a good foundation for the downstream analysis, such as collinearity analysis, eukaryotic genome evolution mechanisms identification, homologous genes isolation, and so on.

The gene family isolation was one of the most important aspects of the gene information mining from genome sequence. *Arabidopsis thaliana* gene sequence was usually used as the query, and search the homologous genes from genome sequence database. In order to test the influence of low quality genome sequence on gene family analysis, we took *Arabidopsis thaliana*, *Vitis vinifera* and *Zea mays* as examples to isolate the homologous genes involved in the anthocyanin biosynthesis pathway, including *Phenylalanine ammonia lyase* (*PAL*), *Cinnamate 4-hydroxylase* (*C4H*), *4*-*coumarate CoA ligase* (*4CL*), *Chalcone synthase* (*CHS*), *Chalcone isomerase* (*CHI*), *Flavanone 3-hydroxylase* (*F3H*), *Flavanone 3'*-*hydroxylase* (*F3’H*), *Flavanone 3'5'*-*hydroxylase* (*F3’5’H*), *Dihydroflavonol reductase* (*DFR*), *Anthocyanidin synthase*/*Leucoanthocyanidin dioxygenase* (*ANS*/*LDOX*), and *UDP*-*flavonoid glucosyl transferase* (*UFGT*).

**Figure 7:**
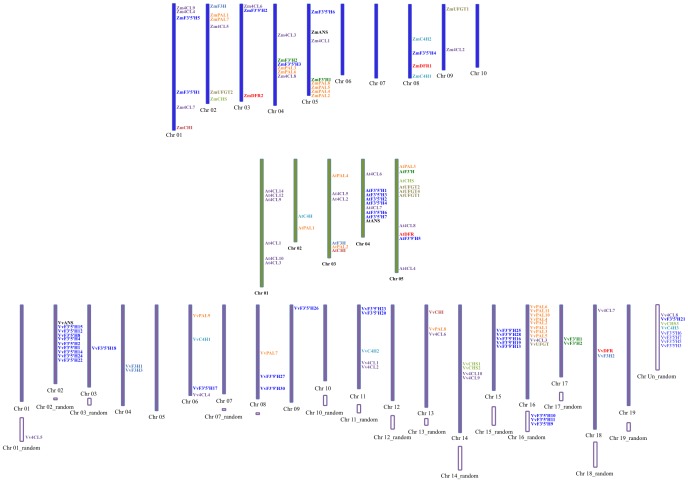
Distribution map of homologous genes involved in anthocyanin biosynthesis pathway in *Arabidopsis thaliana* (middle one), *Vitis vinifera* (up one) and *Zea Mays* (down one).

After Blast search and protein domain analysis, the homologue genes of anthocyanin biosynthesis pathway was isolated and mapped (**Figure** **7**, **Table S5**). *PAL* was the first structural gene in the pathway, and four copies (*AtPAL1*, *AtPAl2*, *AtPAL3*, and *AtPAL4*) were reported in *Arabidopsis thaliana*, eight and eleven copies were isolated from *Zea mays* and *Vitis vinifera*. *AtC4H* (AT2G30490) was located on *Arabidopsis thaliana* chromosome 2, *ZmC4H1* and *ZmC4H2* (GRMZM2G010468 and GRMZM2G139874) was located on *Zea mays* chromosome 8. Three copies of *C4H* in *Vitis vinifera* were isolated, *VvC4H1* and *VvC4H2* (GSVIVP00023932001 and GSVIVP00017017001) were located on *Vitis vinifera* chromosome 6 and 11, respectively, but *VvC4H3* (GSVIVP00007155001) was located on chromosome unknown_random. The result increases the difficulty of collinearity analysis among *Arabidopsis thaliana*, *Vitis vinifera* and *Zea mays*. The similar results were also found in *4CL*, *CHS* and *F3’5’H*. Such as *4CL*, fourteen *At4CLs* were located on chromosome 1, 3, 4 and 5. Nine *Zm4CLs* were located on chromosome 1, 2, 3, 4, 5 and 9. Eight of ten *Vv4CLs* were located on chromosome 6, 11, 13, 14, 16 and 18, while two *Vv4CLs* were located on chromosome unknown_random sequence. Only one *CHS* were identified in *Arabidopsis thaliana* and *Zea mays*, respectively, and three *CHSs* were identified in *Vitis vinifera*. Two of *VvCHSs* were located on chromosome 14, and locations were comparatively close to each other (GSVIVP00037969001: chr14 13875792–13877198, GSVIVP00037967001: chr14 13889324–13890882), while *VvCHS3* was located on chromosome unknown_random. The lack of location information of *VvCHS3* was harmful to the evolution analysis of grape *CHS*, it was unable to estimate the reason of increasing copy number of *VvCHS* was chromosome replication or the combing of chromosome replication and exchange between two chromosomes. Six copies of *AtF3’5’H* were located on chromosome 4, while one copy of *AtF3’5’H* was located on chromosome 5. Two copies of *ZmF3’5’H* were located on chromosome 1, while four copies of *ZmF3’5’H* were located on chromosome 3, 4, 5 and 8. Thirty copies of *VvF3’5’H* were identified in *Vitis vinifera*, but eight copies of *VvF3’5’H* were located on the random sequence, including 3 *VvF3’5’Hs* were located on chromosome 16_random sequence and 5 *VvF3’5’Hs* were located on chromosome unknown_random sequence.

Totally, 66 homologous genes involved in the anthocyanin biosynthesis pathway were identified while twelve genes (18.18%) were located on the random sequences. Eight genes were located on chromosome unknown_random sequence, three genes were located on chromosome 16_random sequence, and one gene was located on chromosome 1_random sequence. The result was observed that a lot of genes were still existed in plant scaffold sequences or unassembled into scaffold sequences and low quality of genome sequence has the influence on genomics downstream analysis, improve the quality of chromosome sequence should be the important task for many plants which have finished the whole genome sequencing project.

## Discussion

Plants form the base of the food chain and are essential model organisms for studying biological systems such as the role of transposons and epigenetics [63]. Whole genome sequencing can provide complete information of the genome structure from the perspective of structural genomics, and thus lay the foundation for the post-genomic era of functional genomics research. For this reason, many researchers are keen to sequence plant genomes and availability of experiment at low cost for second-generation DNA sequencing technologies. Some plant species have been sequenced and more than a dozen are in the pipe line. It is foreseeable that whole-genome sequencing project for a large number of plants will be completed in the near future.

Compared with the advances in animal genome sequencing where by the end of 2012, whole-genome sequencing projects of more than 110 species of animals had been completed (http://en.wikipedia.org/wiki/List_of_sequenced_animal_genomes), the development of plant genome sequencing projects has been limited by a number of factors. 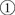 Normally, the genome size of plants are nearly 100 times larger than bird, fish or mammalian genomes [64]–[67]. 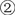 Plants are also of a higher ploidy level (up to 80%), and higher rates of heterozygosity and repeats than others [68], [69]. 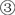 It is difficult to prepare proper libraries for sequencing because it is difficult to extract large quantities of high-quality DNA from plant material [70]. 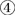 Assembling plant genome requires the proper combination of coverage, read length and quality [53], a high percent of repeat DNA exists in plant genomes, but effective assembly software are not reported.

The large genome sizes, complexity, polyploidy as well as computational and bio-molecular reasons, make *de novo* assembly of plant genome sequences a challenge. In addition to *Arabidopsis thaliana*, *Oryza sativa* and *Zea mays*, which were sequenced using a BAC-by-BAC approach, most of the plant genome sequences were obtained using a WGS (Whole-genome shotgun) approach. There are often time and cost savings of using WGS over a BAC-by-BAC approach; however these benefits are offset by the difficulty in assembling the reads. With large and complex cereal genomes, WGS remains shaky, since majority of the reads will represent repetitive elements in the genome [71], [72].

In contrast to the incredible advancement in throughput, assembling sequencing reads remains a substantial undertaking, much greater than the sequencing efforts alone would suggest [53], [73], [74]. The sequencing process for an appreciable number of plants is already finished, but the whole chromosome sequencing in most of these plants is still unpublished, and even though some plants already have available chromosome sequences, the quality of these sequences is still suspect, especially for plants where *de novo* sequencing strategy was used and have no reference sequence as the assembly template. With the rapid developments in plant genome sequencing, it is necessary for to undertake quality analysis of plant genome sequences, and to provide a reference for researchers and plant genome sequencing project development.

Integrity and accuracy were suggested as parameters for analyzing the quality of genome sequences. Results from this study show that the genome sequence quality for most of the 32 selected plant species was lower than earlier envisaged. The 13 plants with chromosome sequences released were divided into four categories with *Arabidopsis thaliana*, *Oryza sativa* and *Zea mays* belonging to the first level where the integrity, accuracy and the proportion of segments with matching ESTs were higher than the other plants, probably because of their small genome size (*Arabidopsis thaliana* genome size is 125 Mb), sequencing strategy (Sanger method, BAC-by-BAC approach, and many reference sequences), and research intensity (intensive and extensive research has been done on these important model plants, and high quality chromosome sequences produced after repeated modification, 9 times in *Arabidopsis thaliana*, 4 times in *Oryza sativa*). *Brachypodium distachyon*, *Populus trichocarpa*, *Vitis vinifera* and *Glycine max* belonged to the second level, and *Sorghum bicolor*, *Solanum lycopersicum* and *Fragaria vesca* were in the third level. The integrity and accuracy of the second and third level plants was larger than 80%, but the proportion of segments with matching ESTs were lower than in the first level plants. *Lotus japonicus*, *Medicago truncatula* and *Malus* × *domestica* belonged to the last level. From the above analysis, *Lotus japonicas* genome sequence had the lowest quality since despite having high chromosome integrity (CS/GS ratio of 98.09%), the accuracy (mEST/sEST ratio of 52.93%) and the proportion of segments with matching ESTs (12.88%) was the lowest. Comparing genome sequence quality in *Medicago truncatula* and *Lotus japonicas* with that of *Malus* × *domestica* indicates that the integrity of chromosome sequence in the first two plants was 94.48%, and the accuracy of chromosome and the proportion of segments with matching ESTs were 69.61% and 27.39%, respectively, while *Malus* × *domestica* had a contrasting quality. In the SSG plants, the lowest integrity of genome sequence was 55.31% (*Cucumis sativus*), and the highest was 100% (*Arabidopsis lyrata*). The accuracy of genome sequence ranged from 89.02% to 98.85%. These results illustrate that the plant genome can be sequenced in a short time and low cost by the NGS platform and *de novo* strategy, though sequence assembly is still was a big challenge due to the characteristics of plant genomes [71], [72]. Genome sequencing projects for *Physcomitrella patens* and *Carica papaya* were finished in 2008, but till now, the chromosome sequences have not been published. The development of sequencing reads lengths, sequence assemblers and algorithms is slow in comparison with the rapid development of DNA sequencing technology [70], [73], [74].

There are many factors that are influencing the quality of genome sequences. In the analysis of GC content and chromosome sequence quality, we found that there was no influence on genome quality when the GC content was between 30%–50%, but GC content below 30%, influenced on genome sequence quality significantly, as exemplified by *Medicago truncatula* and *Lotus japonicas*. A positive relationship was found between GC content and the proportion of segments with matching ESTs whereby an increase in GC content, led to a rise in the proportion of segments with matching ESTs meaning that the gene density was also increased. This observation agrees with that found in the human genome [67], [75].

Repeat DNA is one of the most important factors for genome sequencing assembly. Previous result have shown that the repetitive DNA content ranges from 64% to 73% in maize [76], 35% to 45% in humans [67], [77] and from 25% in *Oryza coarctata* to 66% in *Oryza officinalis* [78]. The interspersed repeat sequence, low complexity sequence and simple repeat sequence were identified by RepeatMasker software. From the above results, these three types of repeat sequence were shown to have a positive relationship with EST-Chromosome mapping results in some chromosomes, and a negative relationship with EST-Chromosome mapping result in other chromosomes. This indirectly proves that repeat sequences are among the most important factors affecting plant genome sequence quality. Tandemly repeated DNA being repeat sequences, also influence genome sequence quality. Repeat sequences used to be regarded as a challenge in plant genome sequencing, especially in the situation where sequencing reads of NGS platform was short and the proportion of repeat sequences in plant genome was big (repeat sequence was found as 84% in the maize genome sequence and 92% in the rice genome sequence) [19], [21], [72], [79], [80].

The completion of plant genome sequencing projects promote the development of functional genomics, structural genomics, proteomics and metabolomics, especially the rapid development of large-scale gene family identification and collinearity analysis of gene clustes. However, due to the inherent shortcomings of NGS platform and assembler make the chromosome sequence quality of many plants was lower than envisaged. A lot of important gene information was found to exist in the unassembled scaffold sequence or short reads, and these sequences were discarded as “Junk sequences”. Because of the high quality of the human genome sequence can be used as a reference/control in correction of many animal genome sequences. However, chromosome sequence correction work has successful in only few plants, such as *Arabidopsis thaliana* and *Oryza sativa*. The chromosome sequences of many plants are still unreleased despite completion of their genome sequencing projects [80].

In order to improve the genome sequence quality and reduce the influence of poor quality sequences on downstream genomics analysis, the following two points are suggested. First, research on the development of genome sequencing assembler and related algorithms should be increased as earlier suggested by the Pop’s view [81]. The present bioinformatics tools and older algorithms are not capable of accurately assembling the much more complex genomes of organisms. Improved algorithms for accurately assembling complex genomes at scale and improve analytics to record, manipulate analyzed and visualize features for translation of the salient assembly information to the broader community of plant biologists should be encouraged. Secondly, correction work on plants genome sequences should be encouraged since from this study it is discernible that the quality of most plant genome sequences was lower than envisaged, and a lot of important gene information exists in the unassembled sequences. Researchers should focus on the study of “Junk sequences” as it appears to contain a lot of potentially important information. Furthermore, we encourage researchers to open up the genome sequencing data, and with the help of cloud computing and other new technologies, seek new solutions in order to deal with the plant genome sequencing in a much improved manner.

## Supporting Information

Figure S1 **Distribution map of the percentage of segments with matching ESTs in** ***Brachypodium distachyon, Fragaria vesca, Glycine max, Lotus japonicus, Malus x domestica, Medicago truncatula, Oryza sativa, Populus trichocarpa, Solanum lycopersicum*** **, and** ***Sorghum bicolor*** **.** The species name is shown in the end of each chromosome model, and different gradient colors were describes the abundance in each plant. According to blast results, the abundance of EST sequences were divided into six parts, including 0, 1–2, 3–4, 5–6, 7–8, >8 in Oryza sativa, and 0, 1, 2, 3, 4, >4 in others.
(TIF)

Figure S2 **Draft of gene clusters in six plants.** The order was cluster 2, cluster 3, cluster 4, cluster 5, cluster 6, cluster 7, cluster 8, and cluster 10. Cluster 1 and 9 was shown in **Fig. 6**.
(TIF)

Table S1 **Proportions of segments with matching ESTs in the chromosomes of CSG plants.**
(DOC)

Table S2 **GC content in each chromosome of CSG plants.**
(DOC)

Table S3 **Identification of EST location and repeat sequence in thirty regions of six plants.**
(DOC)

Table S4 **Information of ten gene clusters in six plants.**
(DOC)

Table S5 **Information of homologue genes involved in anthocyanin biosynthesis pathway in three plants.**
(DOC)
