# Supplementary figures and images for: Evaluation of Genome Sequencing Quality in Selected Plant Species Using Expressed Sequence Tags
Source: PLoS One. 2013 Jul 29;8(7):e69890. doi: 10.1371/journal.pone.0069890 (PMC3726750; doi:10.1371/journal.pone.0069890)

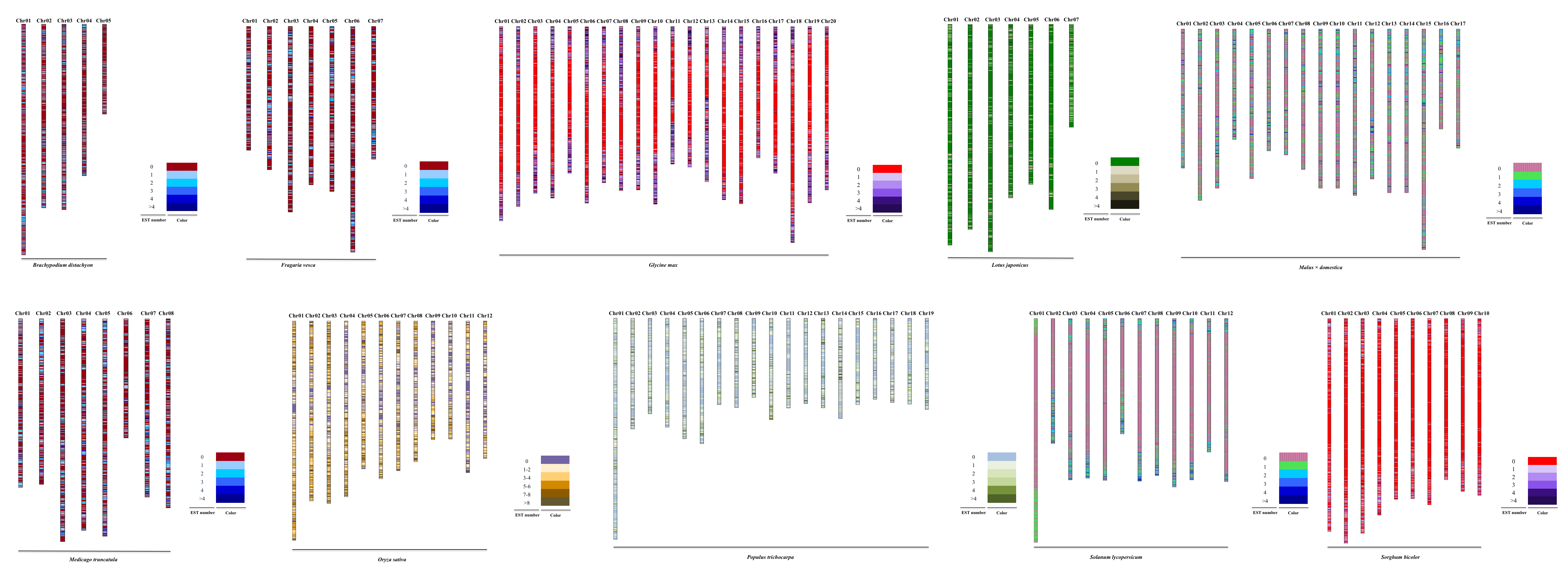

Supplement: Figure S1 — Distribution map of the percentage of segments with matching ESTs in Brachypodium distachyon, Fragaria vesca, Glycine max, Lotus japonicus, Malus x domestica, Medicago truncatula, Oryza sativa, Populus trichocarpa, Solanum lycopersicum , and Sorghum bicolor . The species name is shown in the end of each chromosome model, and different gradient colors were describes the abundance in each plant. According to blast results, the abundance of EST sequences were divided into six parts, including 0, 1–2, 3–4, 5–6, 7–8, >8 in Oryza sativa, and 0, 1, 2, 3, 4, >4 in others. (TIF) [file pone.0069890.s001.tif]

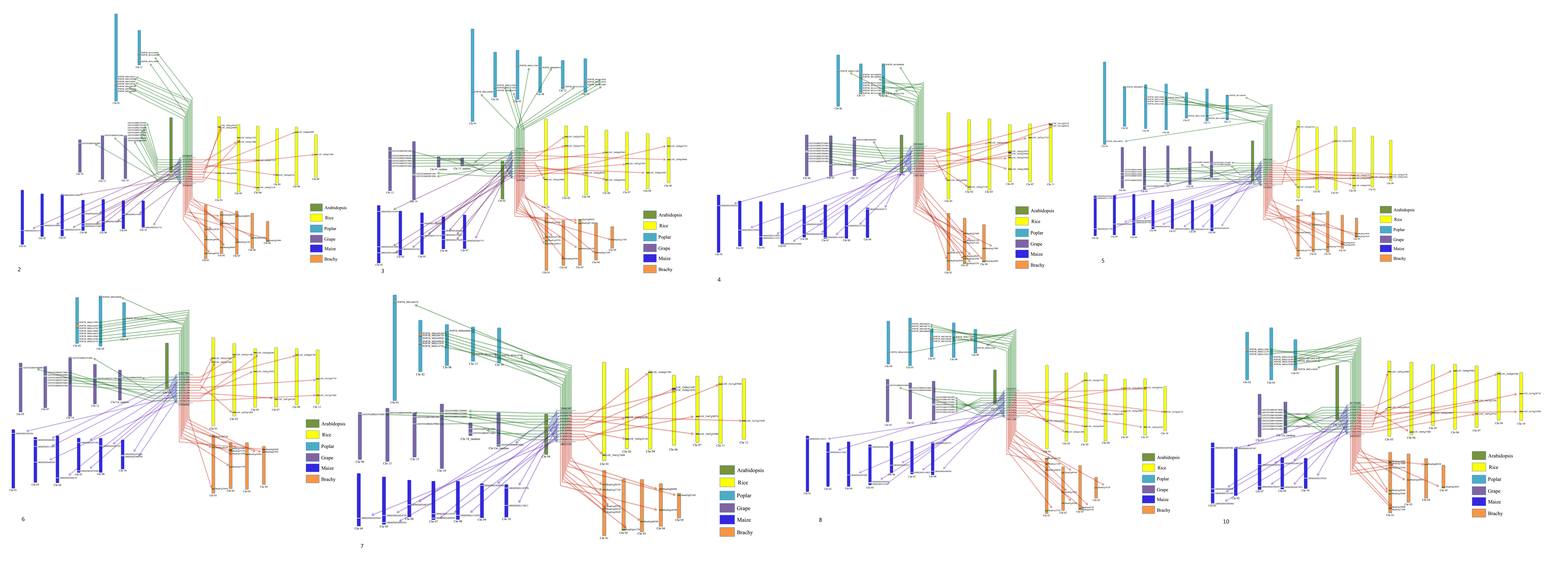

Supplement: Figure S2 — Draft of gene clusters in six plants. The order was cluster 2, cluster 3, cluster 4, cluster 5, cluster 6, cluster 7, cluster 8, and cluster 10. Cluster 1 and 9 was shown in Fig. 6 . (TIF) [file pone.0069890.s002.tif]
