# Supplementary material for: Evaluation of Genome Sequencing Quality in Selected Plant Species Using Expressed Sequence Tags
Source: PLoS One. 2013 Jul 29;8(7):e69890. doi: 10.1371/journal.pone.0069890 (PMC3726750; doi:10.1371/journal.pone.0069890)
Supplement: Table S1 — Proportions of segments with matching ESTs in the chromosomes of CSG plants. (DOC) [file pone.0069890.s003.doc]

**Table S1 Proportions of segments with matching ESTs in the chromosomes of CSG plants**

| **Plant species** | **Chr_1** | **Chr_2** | **Chr_3** | **Chr_4** | **Chr_5** | **Chr_6** | **Chr_7** | **Chr_8** | **Chr_9** | **Chr_10** | **Chr_11** |
| --- | --- | --- | --- | --- | --- | --- | --- | --- | --- | --- | --- |
| *Arabidopsis thaliana* | 93.42% | 93.40% | 90.21% | 93.01% | 90.37% |  |  |  |  |  |  |
| *Brachypodium distachyon* | 35.83% | 35.24% | 35.73% | 29.77% | 25.35% |  |  |  |  |  |  |
| *Fragaria vesca* | 33.81% | 36.33% | 30.84% | 24.26% | 32.75% | 30.79% | 29.65% |  |  |  |  |
| *Glycine max* | 30.77% | 41.78% | 34.94% | 36.79% | 42.00% | 43.98% | 44.30% | 55.11% | 42.52% | 39.41% | 48.21% |
| *Lotus japonicus* | 17.07% | 13.46% | 11.56% | 14.18% | 13.67% | 6.99% |  |  |  |  |  |
| *Malus × domestica* | 31.74% | 28.02% | 30.27% | 43.48% | 33.54% | 34.51% | 26.89% | 27.70% | 31.16% | 33.83% | 33.71% |
| *Medicago truncatula* | 37.35% | 43.87% | 34.09% | 36.90% | 42.36% | 24.68% | 36.36% | 38.50% |  |  |  |
| *Oryza sativa* | 80.49% | 81.25% | 87.13% | 69.92% | 78.33% | 71.96% | 71.38% | 70.88% | 64.71% | 66.67% | 58.44% |
| *Populus trichocarpa* | 54.55% | 55.08% | 51.98% | 48.28% | 49.61% | 56.13% | 46.36% | 49.47% | 67.44% | 62.33% | 38.10% |
| *Solanum lycopersicum* | 22.81% | 29.66% | 25.46% | 19.66% | 16.62% | 28.91% | 20.37% | 18.89% | 18.76% | 17.28% | 19.10% |
| *Sorghum bicolor* | 31.71% | 18.36% | 22.85% | 19.85% | 11.86% | 15.27% | 12.29% | 12.79% | 14.93% | 19.18% |  |
| *Vitis vinifera* | 45.65% | 43.09% | 34.72% | 48.12% | 50.80% | 49.77% | 39.05% | 53.57% | 28.70% | 27.07% | 43.94% |
| *Zea mays* | 95.35% | 96.62% | 96.55% | 97.93% | 98.62% | 98.22% | 96.05% | 99.43% | 97.45% | 99.33% |  |

**Table S1 Proportions of segments with matching ESTs in the chromosomes of CSG plants (continued)**

| **Plant species** | **Chr_12** | **Chr_13** | **Chr_14** | **Chr_15** | **Chr_16** | **Chr_17** | **Chr_18** | **Chr_19** | **Chr_20** | **Average** |
| --- | --- | --- | --- | --- | --- | --- | --- | --- | --- | --- |
| *Arabidopsis thaliana* |  |  |  |  |  |  |  |  |  | 92.03% |
| *Brachypodium distachyon* |  |  |  |  |  |  |  |  |  | 33.49% |
| *Fragaria vesca* |  |  |  |  |  |  |  |  |  | 31.06% |
| *Glycine max* | 42.39% | 59.68% | 32.39% | 39.10% | 41.71% | 44.39% | 34.83% | 37.94% | 38.46% | 41.13% |
| *Lotus japonicus* |  |  |  |  |  |  |  |  |  | 12.88% |
| *Malus × domestica* | 32.18% | 35.45% | 34.93% | 34.96% | 38.65% | 35.34% |  |  |  | 33.40% |
| *Medicago truncatula* |  |  |  |  |  |  |  |  |  | 37.29% |
| *Oryza sativa* | 60.43% |  |  |  |  |  |  |  |  | 72.82% |
| *Populus trichocarpa* | 40.27% | 49.68% | 56.50% | 47.68% | 52.48% | 45.58% | 53.33% | 37.50% |  | 51.18% |
| *Solanum lycopersicum* | 15.88% |  |  |  |  |  |  |  |  | 20.83% |
| *Sorghum bicolor* |  |  |  |  |  |  |  |  |  | 18.31% |
| *Vitis vinifera* | 41.41% | 35.25% | 36.30% | 28.08% | 33.03% | 46.78% | 42.86% | 36.25% |  | 40.34% |
| *Zea mays* |  |  |  |  |  |  |  |  |  | 97.38% |
