# Supplementary material for: Evaluation of Genome Sequencing Quality in Selected Plant Species Using Expressed Sequence Tags
Source: PLoS One. 2013 Jul 29;8(7):e69890. doi: 10.1371/journal.pone.0069890 (PMC3726750; doi:10.1371/journal.pone.0069890)
Supplement: Table S2 — GC content in each chromosome of CSG plants. (DOC) [file pone.0069890.s004.doc]

**Table S2 GC content in each chromosome of CSG plants**

| **Plant species** | **Chr_1** | **Chr_2** | **Chr_3** | **Chr_4** | **Chr_5** | **Chr_6** | **Chr_7** | **Chr_8** | **Chr_9** | **Chr_10** | **Chr_11** |
| --- | --- | --- | --- | --- | --- | --- | --- | --- | --- | --- | --- |
| *Arabidopsis thaliana* | 35.68% | 35.86% | 36.32% | 36.20% | 35.93% |  |  |  |  |  |  |
| *Brachypodium distachyon* | 46.09% | 46.29% | 46.15% | 45.98% | 46.83% |  |  |  |  |  |  |
| *Fragaria vesca* | 35.77% | 36.07% | 35.42% | 35.40% | 35.96% | 35.72% | 36.34% |  |  |  |  |
| *Glycine max* | 34.93% | 34.24% | 34.02% | 34.09% | 32.99% | 34.96% | 34.20% | 34.00% | 33.91% | 34.38% | 34.62% |
| *Lotus japonicus* | 14.07% | 10.48% | 8.86% | 11.49% | 11.17% | 5.77% |  |  |  |  |  |
| *Malus × domestica* | 25.99% | 27.87% | 27.16% | 27.74% | 27.32% | 27.59% | 27.76% | 27.77% | 28.74% | 27.87% | 27.46% |
| *Medicago truncatula* | 25.10% | 28.50% | 27.00% | 26.44% | 32.03% | 24.25% | 28.14% | 25.88% |  |  |  |
| *Oryza sativa* | 42.03% | 42.31% | 42.59% | 43.66% | 43.69% | 42.39% | 42.59% | 43.24% | 41.87% | 42.09% | 39.62% |
| *Populus trichocarpa* | 32.74% | 33.20% | 32.49% | 32.64% | 32.60% | 32.63% | 32.76% | 33.99% | 33.67% | 33.86% | 31.92% |
| *Solanum lycopersicum* | 32.15% | 31.80% | 31.86% | 31.77% | 31.99% | 32.12% | 32.35% | 32.30% | 32.67% | 31.82% | 32.07% |
| *Sorghum bicolor* | 40.64% | 42.98% | 43.08% | 40.54% | 41.29% | 43.00% | 40.89% | 40.82% | 41.01% | 40.91% |  |
| *Vitis vinifera* | 33.77% | 33.87% | 33.80% | 33.64% | 34.08% | 33.36% | 33.61% | 34.09% | 32.88% | 33.36% | 34.03% |
| *Zea mays* | 46.67% | 46.66% | 46.60% | 46.29% | 46.61% | 46.82% | 46.47% | 46.66% | 46.68% | 46.64% |  |

**Table S2 GC content in each chromosome of CSG plants** (continued)

| **Plant species** | **Chr_12** | **Chr_13** | **Chr_14** | **Chr_15** | **Chr_16** | **Chr_17** | **Chr_18** | **Chr_19** | **Chr_20** | **Average** |
| --- | --- | --- | --- | --- | --- | --- | --- | --- | --- | --- |
| *Arabidopsis thaliana* |  |  |  |  |  |  |  |  |  | 35.97% |
| *Brachypodium distachyon* |  |  |  |  |  |  |  |  |  | 46.21% |
| *Fragaria vesca* |  |  |  |  |  |  |  |  |  | 35.78% |
| *Glycine max* | 34.28% | 34.64% | 33.78% | 34.91% | 34.46% | 33.69% | 33.97% | 33.61% | 34.15% | 34.21% |
| *Lotus japonicus* |  |  |  |  |  |  |  |  |  | 10.34% |
| *Malus × domestica* | 27.45% | 28.19% | 26.95% | 27.20% | 28.15% | 28.61% |  |  |  | 27.62% |
| *Medicago truncatula* |  |  |  |  |  |  |  |  |  | 27.39% |
| *Oryza sativa* | 42.77% |  |  |  |  |  |  |  |  | 42.41% |
| *Populus trichocarpa* | 32.79% | 32.49% | 33.56% | 32.73% | 32.80% | 32.59% | 32.34% | 32.22% |  | 32.84% |
| *Solanum lycopersicum* | 32.31% |  |  |  |  |  |  |  |  | 32.11% |
| *Sorghum bicolor* |  |  |  |  |  |  |  |  |  | 41.56% |
| *Vitis vinifera* | 34.02% | 33.60% | 33.83% | 33.63% | 32.86% | 34.20% | 33.92% | 33.21% |  | 33.67% |
| *Zea mays* |  |  |  |  |  |  |  |  |  | 46.60% |
