# Supplementary material for: Evaluation of Genome Sequencing Quality in Selected Plant Species Using Expressed Sequence Tags
Source: PLoS One. 2013 Jul 29;8(7):e69890. doi: 10.1371/journal.pone.0069890 (PMC3726750; doi:10.1371/journal.pone.0069890)
Supplement: Table S3 — Identification of EST location and repeat sequence in thirty regions of six plants. (DOC) [file pone.0069890.s005.doc]

**Table S3 Identification of EST location and repeat sequence in thirty regions of six plants**

| **Species** | **Position** | **EST Number** | **Repeat DNA Number/Length (bp)** | **Position** | **EST Number** | **Repeat DNA Number/Length (bp)** | **Position** | **EST Number** | **Repeat DNA Number/Length (bp)** |
| --- | --- | --- | --- | --- | --- | --- | --- | --- | --- |
| *Arabidopsis thaliana* | Chr 1_0.2S | 1,726 | 28/1,399 | Chr 2_0.1S | 975 | 13/770 | Chr 4_0.1S | 1,251 | 53/2,593 |
|  | Chr 1_2.6S | 599 | 18/735 | Chr 2_0.2S | 908 | 75/3,395 | Chr 4_0.2S | 758 | 14/499 |
|  | Chr 1_5.1S | 884 | 21/1,252 | Chr 2_1.9S | 1,085 | 72/3,684 | Chr 4_0.6S | 505 | 81/4,269 |
|  | Chr 1_13.6S | 183 | 52/3,426 | Chr 2_2.5S | 1,489 | 51/3,290 | Chr 4_0.7S | 1,137 | 53/2,570 |
|  | Chr 1_15.0S | 173 | 29/3,112 | Chr 2_2.7S | 606 | 40/1,933 | Chr 4_1.0S | 400 | 39/1,570 |
|  | Chr 1_15.2S | 2 | 84/12,258 | Chr 2_3.2S | 373 | 12/1,248 | Chr 4_1.5S | 462 | 42/2,291 |
|  | Chr 1_15.4S | 69 | 17/2,003 | Chr 2_4.6S | 161 | 25/1,529 | Chr 4_2.7S | 1,815 | 42/2,388 |
|  | Chr 1_16.2S | 465 | 74/4,807 | Chr 2_5.3S | 29 | 31/1,854 | Chr 4_7.8S | 436 | 85/4,196 |
|  | Chr 1_16.4S | 163 | 75/4,375 | Chr 2_19.0S | 853 | 24/953 | Chr 4_13.5S | 956 | 21/804 |
|  | Chr 1_22.0S | 483 | 52/2,508 | Chr 2_19.3S | 1,733 | 49/1,875 | Chr 4_15.2S | 702 | 21/1,021 |
| *Brachypodium distachyon* | Chr 2_1.8S | 44 | 15/731 | Chr 4_0.1S | 161 | 20/1,301 | Chr 5_0.1S | 0 | 89/56,973 |
|  | Chr 2_12.3S | 21 | 24/1,607 | Chr 4_1.1S | 58 | 20/762 | Chr 5_0.4S | 45 | 15/636 |
|  | Chr 2_14.6S | 88 | 18/990 | Chr 4_2.5S | 43 | 14/661 | Chr 5_1.5S | 2 | 25/1,652 |
|  | Chr 2_19.8S | 4 | 20/1,125 | Chr 4_10.5S | 3 | 23/1,594 | Chr 5_2.5S | 68 | 17/688 |
|  | Chr 2_20.0S | 12 | 24/1,404 | Chr 4_10.8S | 4 | 27/1,427 | Chr 5_3.6S | 2 | 12/818 |
|  | Chr 2_28.4S | 3 | 23/1,078 | Chr 4_11.0S | 16 | 36/3,413 | Chr 5_5.9S | 6 | 14/713 |
|  | Chr 2_34.9S | 52 | 10/583 | Chr 4_16.6S | 29 | 18/1,003 | Chr 5_9.6S | 96 | 12/647 |
|  | Chr 2_35.6S | 0 | 26/1,495 | Chr 4_17.3S | 6 | 19/754 | Chr 5_10.8S | 51 | 8/479 |
|  | Chr 2_43.8S | 67 | 11/530 | Chr 4_44.7S | 84 | 11/609 | Chr 5_10.9S | 0 | 21/1,263 |
|  | Chr 2_49.3S | 42 | 5/268 | Chr 4_45.3S | 50 | 13/917 | Chr 5_26.4S | 85 | 15/524 |
| *Glycine max* | Chr 4_0.9S | 1,001 | 171/10,118 | Chr10_0.4S | 158 | 172/10,274 | Chr16_1.1S | 310 | 138/7,844 |
|  | Chr 4_3.1S | 161 | 181/12,021 | Chr10_2.1S | 410 | 97/5,814 | Chr16_1.3S | 840 | 140/8,599 |
|  | Chr 4_9.1S | 575 | 146/8,877 | Chr10_11.2S | 21 | 50/3,525 | Chr16_7.6S | 158 | 114/6,744 |
|  | Chr 4_15.6S | 93 | 79/4,160 | Chr10_18.9S | 150 | 87/6,168 | Chr16_18.2S | 2 | 30/2,190 |
|  | Chr 4_18.1S | 39 | 39/2,082 | Chr10_26.8S | 6 | 22/1,745 | Chr16_18.8S | 251 | 81/6,056 |
|  | Chr 4_19.6S | 5 | 48/3,346 | Chr10_27.3S | 33 | 34/2,278 | Chr16_19.4S | 13 | 107/6,904 |
|  | Chr 4_26.4S | 12 | 44/3,201 | Chr10_32.8S | 67 | 61/3,586 | Chr16_19.5S | 77 | 94/5,393 |
|  | Chr 4_27.9S | 35 | 47/3,294 | Chr10_33.4S | 82 | 52/3,704 | Chr16_20.1S | 121 | 46/2,663 |
|  | Chr 4_28.0S | 111 | 71/5,192 | Chr10_37.5S | 255 | 94/5,335 | Chr16_20.7S | 5 | 23/1,416 |
|  | Chr 4_41.4S | 213 | 119/6,877 | Chr10_49.6S | 258 | 146/9,099 | Chr16_22.5S | 20 | 22/1,265 |

**Table S3 Identification of EST location and repeat sequence in thirty regions of six plants** (continued)

| **Species** | **Position** | **EST Number** | **Repeat DNA Number/Length (bp)** | **Position** | **EST Number** | **Repeat DNA Number/Length (bp)** | **Position** | **EST Number** | **Repeat DNA Number/Length (bp)** |
| --- | --- | --- | --- | --- | --- | --- | --- | --- | --- |
| *Oryza sativa* | Chr 2_1.0S | 613 | 60/3,164 | Chr 7_0.7S | 786 | 49/2,187 | Chr11_0S | 542 | 40/2,016 |
|  | Chr 2_4.2S | 300 | 73/4,376 | Chr 7_2.1S | 839 | 49/2,580 | Chr11_1.4S | 759 | 40/2,158 |
|  | Chr 2_4.9S | 327 | 66/4,288 | Chr 7_5.5S | 264 | 62/4,047 | Chr11_3.3S | 599 | 47/2,285 |
|  | Chr 2_7.0S | 496 | 53/3,170 | Chr 7_8.7S | 96 | 54/4,212 | Chr11_10.2S | 78 | 28/2,408 |
|  | Chr 2_11.9S | 432 | 53/2,875 | Chr 7_14.3S | 66 | 34/1,863 | Chr11_10.3S | 55 | 47/2,379 |
|  | Chr 2_14.4S | 41 | 5/393 | Chr 7_14.8S | 419 | 36/3,313 | Chr11_16.8S | 107 | 52/4,075 |
|  | Chr 2_14.6S | 282 | 46/2,706 | Chr 7_16.5S | 170 | 39/2,105 | Chr11_19.8S | 70 | 42/1,889 |
|  | Chr 2_21.2S | 49 | 33/2,159 | Chr 7_18.8S | 218 | 61/4,281 | Chr11_21.9S | 347 | 48/2,631 |
|  | Chr 2_23.3S | 268 | 70/4,470 | Chr 7_27.1S | 369 | 37/1,453 | Chr11_28.6S | 86 | 46/4,100 |
|  | Chr 2_26.6S | 259 | 52/3,487 | Chr 7_28.7S | 389 | 37/2,558 | Chr11_30.7S | 2,587 | 55/3,095 |
| *Vitis vinifera* | Chr 1_0.5S | 272 | 19/1,930 | Chr 7_0.4S | 1,446 | 45/1,963 | Chr13_0.1S | 576 | 73/4,839 |
|  | Chr 1_1.2S | 114 | 111/6,954 | Chr 7_1.1S | 139 | 108/7,144 | Chr13_1.7S | 92 | 115/7,245 |
|  | Chr 1_3.3S | 287 | 101/8,015 | Chr 7_2.6S | 35 | 109/9,062 | Chr13_2.5S | 607 | 114/7,406 |
|  | Chr 1_6.3S | 78 | 87/4,237 | Chr 7_3.3S | 795 | 136/9,509 | Chr13_4.2S | 224 | 157/9,937 |
|  | Chr 1_8.0S | 147 | 160/10,465 | Chr 7_4.6S | 1,328 | 97/6,272 | Chr13_12.4S | 32 | 47/2,274 |
|  | Chr 1_13.6S | 78 | 69/5,385 | Chr 7_5.5S | 13 | 96/5,172 | Chr13_12.6S | 50 | 19/1,053 |
|  | Chr 1_14.5S | 51 | 39/1,890 | Chr 7_7.1S | 11 | 92/6,327 | Chr13_16.3S | 53 | 72/4,510 |
|  | Chr 1_17.7S | 8 | 82/5,005 | Chr 7_7.7S | 93 | 88/5,517 | Chr13_17.8S | 7 | 92/5,401 |
|  | Chr 1_18.2S | 38 | 72/4,314 | Chr 7_19.4S | 35 | 78/4,033 | Chr13_19.0S | 71 | 151/9,834 |
|  | Chr 1_20.9S | 433 | 200/13,398 | Chr 7_20.5S | 108 | 165/9,671 | Chr13_21.6S | 1,351 | 84/5,303 |
| *Zea mays* | Chr 1_0.2S | 132 | 18/846 | Chr 5_0.7S | 137 | 9/334 | Chr 9_11.2S | 379 | 23/1,016 |
|  | Chr 1_1.1S | 219 | 14/1,108 | Chr 5_3.3S | 230 | 15/697 | Chr 9_13.1S | 173 | 12/572 |
|  | Chr 1_3.5S | 46 | 15/1,327 | Chr 5_20.5S | 93 | 5/172 | Chr 9_35.1S | 5 | 25/1,387 |
|  | Chr 1_7.0S | 224 | 21/1,151 | Chr 5_35.6S | 76 | 18/905 | Chr 9_70.2S | 109 | 10/354 |
|  | Chr 1_7.3S | 293 | 26/1,758 | Chr 5_47.8S | 436 | 6/215 | Chr 9_73.8S | 74 | 13/818 |
|  | Chr 1_53.6S | 836 | 23/1,223 | Chr 5_70.8S | 163 | 28/1,505 | Chr 9_98.4S | 117 | 20/985 |
|  | Chr 1_74.9S | 43 | 16/710 | Chr 5_112.3S | 65 | 15/925 | Chr 9_148.5S | 38 | 8/504 |
|  | Chr 1_248S | 62 | 10/528 | Chr 5_139.6S | 169 | 23/1352 | Chr 9_150.9S | 165 | 19/815 |
|  | Chr 1_277.3S | 338 | 12/393 | Chr 5_174.9S | 49 | 5/217 | Chr 9_151.6S | 756 | 33/1,368 |
|  | Chr 1_281.6S | 236 | 20/609 | Chr 5_200.0S | 59 | 4/159 | Chr 9_153.9S | 3,567 | 14/645 |
