# Supplementary material for: Evaluation of Genome Sequencing Quality in Selected Plant Species Using Expressed Sequence Tags
Source: PLoS One. 2013 Jul 29;8(7):e69890. doi: 10.1371/journal.pone.0069890 (PMC3726750; doi:10.1371/journal.pone.0069890)
Supplement: Table S4 — Information of ten gene clusters in six plants. (DOC) [file pone.0069890.s006.doc]

**Table S4 Information of ten gene clusters in six plants**

| Arabidopsis gene ID | Chr | Start | End | Rice gene ID | Chr | Start | End | Grape gene ID | Chr | Start | End |
| --- | --- | --- | --- | --- | --- | --- | --- | --- | --- | --- | --- |
| AT1G03130 | Chr1 | 753307 | 754198 | LOC_Os08g44680 | Chr8 | 28083293 | 28084298 | GSVIVG00023726001 | Chr7 | 1532531 | 1533319 |
| AT1G03140 | Chr1 | 754371 | 756303 | LOC_Os07g38420 | Chr7 | 23087564 | 23090921 | GSVIVG00023725001 | Chr7 | 1533793 | 1539485 |
| AT1G03150 | Chr1 | 756414 | 758612 | LOC_Os03g49230 | Chr3 | 28039346 | 28042378 | GSVIVG00023724001 | Chr7 | 1540874 | 1572206 |
| AT1G03160 | Chr1 | 761282 | 766158 | LOC_Os05g32390 | Chr5 | 18891981 | 18900862 | GSVIVG00023669001 | Chr7 | 2085435 | 2102387 |
| AT1G03180 | Chr1 | 773411 | 775344 | LOC_Os05g16250 | Chr5 | 9188371 | 9192391 | GSVIVG00023711001 | Chr7 | 1728784 | 1733964 |
| AT1G03190 | Chr1 | 775527 | 780062 | LOC_Os05g05260 | Chr5 | 2584798 | 2590120 | GSVIVG00023710001 | Chr7 | 1735894 | 1744982 |
| AT1G03220 | Chr1 | 787122 | 788651 | LOC_Os05g33400 | Chr5 | 19631962 | 19633443 | GSVIVG00023703001 | Chr7 | 1814105 | 1815415 |
| AT1G03250 | Chr1 | 793353 | 795368 | LOC_Os11g04990 | Chr11 | 2145222 | 2148114 | GSVIVG00023695001 | Chr7 | 1858572 | 1863351 |
| AT1G03280 | Chr1 | 803035 | 807066 | LOC_Os08g25460 | Chr8 | 15494238 | 15501850 | GSVIVG00031472001 | Chr11 | 13924509 | 13927525 |
| AT1G03310 | Chr1 | 813471 | 816749 | LOC_Os05g32710 | Chr5 | 19154970 | 19157600 | GSVIVG00023681001 | Chr7 | 1996730 | 1999378 |
| AT1G78560 | Chr1 | 29546605 | 29548824 | LOC_Os04g31210 | Chr4 | 18675930 | 18681077 | GSVIVG00027925001 | Chr19 | 263260 | 265164 |
| AT1G78570 | Chr1 | 29549649 | 29552438 | LOC_Os03g17000 | Chr3 | 9445273 | 9448703 | GSVIVG00027921001 | Chr19 | 287025 | 290971 |
| AT1G78580 | Chr1 | 29552340 | 29560912 | LOC_Os05g44210 | Chr5 | 25713720 | 25722089 | GSVIVG00027920001 | Chr19 | 292026 | 305463 |
| AT1G78590 | Chr1 | 29564249 | 29566375 | LOC_Os09g17680 | Chr9 | 10810135 | 10812498 | GSVIVG00027917001 | Chr19 | 348042 | 351608 |
| AT1G78600 | Chr1 | 29567187 | 29568859 | LOC_Os06g05890 | Chr6 | 2695460 | 2699503 | GSVIVG00027915001 | Chr19 | 356871 | 371395 |
| AT1G78630 | Chr1 | 29575956 | 29577658 | LOC_Os01g54540 | Chr1 | 31367015 | 31369416 | GSVIVG00027910001 | Chr19 | 417703 | 423612 |
| AT1G78650 | Chr1 | 29582929 | 29585641 | LOC_Os01g10690 | Chr1 | 5718883 | 5724184 | GSVIVG00027850001 | Chr19 | 1027300 | 1038286 |
| AT1G78690 | Chr1 | 29596655 | 29598629 | LOC_Os04g57150 | Chr4 | 34054300 | 34058149 | GSVIVG00029235001 | Chr13 | 2763 | 12542 |
| AT1G78700 | Chr1 | 29599349 | 29601682 | LOC_Os01g10610 | Chr1 | 5668988 | 5671470 | GSVIVG00035819001 | Chr10 | 2102701 | 2106297 |
| AT1G78770 | Chr1 | 29616919 | 29621447 | LOC_Os03g13370 | Chr3 | 7211429 | 7221257 | GSVIVG00027833001 | Chr19 | 1298875 | 1315920 |
| AT2G04520 | Chr2 | 1574592 | 1575863 | LOC_Os02g19770 | Chr2 | 11568248 | 11569114 | GSVIVG00026971001 | Chr15_random | 3483584 | 3484356 |
| AT2G04530 | Chr2 | 1576709 | 1578741 | LOC_Os09g30466 | Chr9 | 18565965 | 18569125 | GSVIVG00025981001 | Chr12 | 6239567 | 6244765 |
| AT2G04540 | Chr2 | 1581416 | 1584688 | LOC_Os02g10320 | Chr2 | 5422121 | 5424926 | GSVIVG00025980001 | Chr12 | 6245293 | 6253506 |
| AT2G04550 | Chr2 | 1588360 | 1590166 | LOC_Os06g20340 | Chr6 | 11684180 | 11687915 | GSVIVG00025979001 | Chr12 | 6255639 | 6269366 |
| AT2G04560 | Chr2 | 1590306 | 1592667 | LOC_Os01g54900 | Chr1 | 31568908 | 31575611 | GSVIVG00025975001 | Chr12 | 6293430 | 6298397 |
| AT2G04570 | Chr2 | 1594673 | 1596349 | LOC_Os06g43044 | Chr6 | 25870091 | 25876328 | GSVIVG00025972001 | Chr12 | 6323932 | 6326218 |
| AT2G04620 | Chr2 | 1610506 | 1612902 | LOC_Os08g32650 | Chr8 | 20219069 | 20221985 | GSVIVG00020819001 | Chr14 | 17482347 | 17484551 |
| AT2G04630 | Chr2 | 1618936 | 1620336 | LOC_Os07g27930 | Chr7 | 16289922 | 16293446 | GSVIVG00018833001 | Chr12 | 1802579 | 1806905 |
| AT2G04660 | Chr2 | 1624741 | 1629162 | LOC_Os04g40830 | Chr4 | 24229225 | 24236905 | GSVIVG00036925001 | Chr1_random | 1221230 | 1231107 |
| AT2G04700 | Chr2 | 1646807 | 1648560 | LOC_Os09g07570 | Chr9 | 3792262 | 3796371 | GSVIVG00020811001 | Chr14 | 17379776 | 17386390 |
| AT2G30300 | Chr2 | 12919401 | 12921222 | LOC_Os05g39800 | Chr5 | 23386717 | 23389211 | GSVIVG00023976001 | Chr6 | 8409729 | 8411940 |
| AT2G30320 | Chr2 | 12925728 | 12927896 | LOC_Os03g21980 | Chr3 | 12591671 | 12595636 | GSVIVG00023973001 | Chr6 | 8431692 | 8436761 |
| AT2G30330 | Chr2 | 12928767 | 12929737 | LOC_Os11g02110 | Chr11 | 573467 | 574724 | GSVIVG00023968001 | Chr6 | 8479853 | 8480984 |
| AT2G30340 | Chr2 | 12931219 | 12932582 | LOC_Os01g60960 | Chr1 | 35257561 | 35259448 | GSVIVG00023965001 | Chr6 | 8516320 | 8517372 |
| AT2G30350 | Chr2 | 12934150 | 12935773 | LOC_Os07g12710 | Chr7 | 7258614 | 7260429 | GSVIVG00023960001 | Chr6 | 8540983 | 8543040 |
| AT2G30390 | Chr2 | 12951027 | 12954090 | LOC_Os05g29760 | Chr5 | 17209580 | 17216776 | GSVIVG00019218001 | Chr7 | 15173356 | 15192903 |
| AT2G30410 | Chr2 | 12959289 | 12960810 | LOC_Os02g57150 | Chr2 | 35002742 | 35004795 | GSVIVG00019045001 | Chr15 | 1788098 | 1794791 |
| AT2G30490 | Chr2 | 12993663 | 12995770 | LOC_Os05g25640 | Chr5 | 14899987 | 14904546 | GSVIVG00023932001 | Chr6 | 8813071 | 8816574 |
| AT2G30520 | Chr2 | 13002742 | 13005600 | LOC_Os11g02610 | Chr11 | 823417 | 825831 | GSVIVG00023923001 | Chr6 | 8947497 | 8949902 |
| AT2G30530 | Chr2 | 13008916 | 13011063 | LOC_Os05g25070 | Chr5 | 14510333 | 14514238 | GSVIVG00023914001 | Chr6 | 9051824 | 9065081 |
| AT3G51140 | Chr3 | 18997600 | 18999904 | LOC_Os03g15033 | Chr3 | 8207583 | 8211913 | GSVIVG00011470001 | ChrUn_random | 70549376 | 70551638 |
| AT3G51240 | Chr3 | 19025264 | 19026939 | LOC_Os04g56700 | Chr4 | 33809347 | 33812300 | GSVIVG00036784001 | Chr4 | 14790912 | 14792727 |
| AT3G51270 | Chr3 | 19033568 | 19036772 | LOC_Os01g66520 | Chr1 | 38631343 | 38635651 | GSVIVG00034779001 | Chr5 | 23242271 | 23253404 |
| AT3G51280 | Chr3 | 19037140 | 19038951 | LOC_Os09g36740 | Chr9 | 21197503 | 21199723 | GSVIVG00036751001 | Chr4 | 15115370 | 15117081 |
| AT3G51290 | Chr3 | 19039980 | 19042569 | LOC_Os09g36760 | Chr9 | 21208855 | 21213168 | GSVIVG00036746001 | Chr4 | 15142325 | 15145742 |
| AT3G51390 | Chr3 | 19075576 | 19078120 | LOC_Os04g47410 | Chr4 | 28131106 | 28136785 | GSVIVG00011882001 | ChrUn_random | 78237608 | 78255039 |
| AT3G51480 | Chr3 | 19100676 | 19104825 | LOC_Os04g49570 | Chr4 | 29565558 | 29570387 | GSVIVG00019331001 | Chr7 | 13435679 | 13439842 |
| AT3G51510 | Chr3 | 19109062 | 19110051 | LOC_Os05g41190 | Chr5 | 24127105 | 24128452 | GSVIVG00008701001 | Chr4 | 16297522 | 16300951 |
| AT3G51520 | Chr3 | 19110596 | 19112760 | LOC_Os02g48350 | Chr2 | 29598974 | 29601846 | GSVIVG00005983001 | Chr4 | 19153302 | 19166807 |
| AT3G51730 | Chr3 | 19186666 | 19188691 | LOC_Os01g07250 | Chr1 | 3422011 | 3425559 | GSVIVG00037466001 | Chr8 | 9264282 | 9267172 |
| AT3G61690 | Chr3 | 22827884 | 22834048 | LOC_Os03g11240 | Chr3 | 5779594 | 5788833 | GSVIVG00006505001 | ChrUn_random | 39408060 | 39419498 |
| AT3G62000 | Chr3 | 22959201 | 22961209 | LOC_Os08g05790 | Chr8 | 3101979 | 3105447 | GSVIVG00026213001 | Chr15 | 7003556 | 7007188 |
| AT3G62080 | Chr3 | 22986174 | 22988777 | LOC_Os01g18280 | Chr1 | 10262946 | 10267962 | GSVIVG00028226001 | Chr7 | 5217105 | 5225789 |
| AT3G62110 | Chr3 | 22997031 | 23000041 | LOC_Os03g61800 | Chr3 | 35037354 | 35041627 | GSVIVG00028234001 | Chr7 | 5268820 | 5272556 |
| AT3G62120 | Chr3 | 23001030 | 23004321 | LOC_Os12g25710 | Chr12 | 14897145 | 14904687 | GSVIVG00028238001 | Chr7 | 5298497 | 5305679 |
| AT3G62140 | Chr3 | 23007083 | 23008749 | LOC_Os01g21860 | Chr1 | 12264911 | 12269085 | GSVIVG00028241001 | Chr7 | 5322061 | 5327488 |
| AT3G62200 | Chr3 | 23023437 | 23025732 | LOC_Os07g44320 | Chr7 | 26474146 | 26478328 | GSVIVG00022522001 | Chr14 | 378687 | 382285 |
| AT3G62240 | Chr3 | 23033442 | 23036765 | LOC_Os05g19970 | Chr5 | 11660556 | 11666381 | GSVIVG00028188001 | Chr7 | 4939045 | 4944245 |
| AT3G62260 | Chr3 | 23038290 | 23040584 | LOC_Os05g04360 | Chr5 | 1994243 | 1996903 | GSVIVG00028179001 | Chr7 | 4882865 | 4885246 |
| AT3G62270 | Chr3 | 23042284 | 23046048 | LOC_Os12g37840 | Chr12 | 23248824 | 23253345 | GSVIVG00019680001 | Chr5 | 2260818 | 2265804 |
| AT4G08550 | Chr4 | 5444345 | 5446891 | LOC_Os06g12030 | Chr6 | 6440413 | 6447606 | GSVIVG00015289001 | Chr18 | 3858328 | 3862342 |
| AT4G08790 | Chr4 | 5608240 | 5611215 | LOC_Os12g31830 | Chr12 | 19151192 | 19156207 | GSVIVG00029495001 | Chr13 | 1935976 | 1946762 |
| AT4G08810 | Chr4 | 5616052 | 5617939 | LOC_Os07g43990 | Chr7 | 26298300 | 26300627 | GSVIVG00024793001 | Chr6 | 749666 | 751342 |
| AT4G08900 | Chr4 | 5703347 | 5705346 | LOC_Os04g01590 | Chr4 | 396481 | 400947 | GSVIVG00026399001 | Chr15 | 5106262 | 5111988 |
| AT4G08920 | Chr4 | 5724103 | 5727253 | LOC_Os02g36380 | Chr2 | 21976509 | 21980296 | GSVIVG00015232001 | Chr18 | 4379229 | 4386017 |
| AT4G08960 | Chr4 | 5746289 | 5748285 | LOC_Os06g11640 | Chr6 | 6171585 | 6176329 | GSVIVG00015198001 | Chr18 | 4618655 | 4622464 |
| AT4G08980 | Chr4 | 5758105 | 5760558 | LOC_Os02g52130 | Chr2 | 31908532 | 31911697 | GSVIVG00009819001 | ChrUn_random | 148512352 | 148513792 |
| AT4G09140 | Chr4 | 5816942 | 5821037 | LOC_Os01g72880 | Chr1 | 42268880 | 42274913 | GSVIVG00038710001 | Chr18_random | 4666396 | 4677300 |
| AT4G09320 | Chr4 | 5923400 | 5924532 | LOC_Os07g30970 | Chr7 | 18334872 | 18337487 | GSVIVG00019061001 | Chr15 | 2121331 | 2123508 |
| AT4G09570 | Chr4 | 6049517 | 6052335 | LOC_Os11g07040 | Chr11 | 3495218 | 3499974 | GSVIVG00031812001 | Chr18_random | 2620478 | 2642088 |
| AT4G39120 | Chr4 | 18225575 | 18227928 | LOC_Os07g09330 | Chr7 | 4890264 | 4893669 | GSVIVG00036332001 | Chr3 | 3155745 | 3160612 |
| AT4G39280 | Chr4 | 18281444 | 18284900 | LOC_Os10g26130 | Chr10 | 13537545 | 13542463 | GSVIVG00036385001 | Chr3 | 3632498 | 3639925 |
| AT4G39370 | Chr4 | 18306238 | 18308842 | LOC_Os04g34984 | Chr4 | 21263959 | 21268754 | GSVIVG00019361001 | Chr7 | 13130660 | 13135362 |
| AT4G39400 | Chr4 | 18324661 | 18328826 | LOC_Os01g52050 | Chr1 | 29927543 | 29931487 | GSVIVG00019364001 | Chr7 | 13091623 | 13095566 |
| AT4G39460 | Chr4 | 18355993 | 18358893 | LOC_Os05g29860 | Chr5 | 17276735 | 17280614 | GSVIVG00019370001 | Chr7 | 12999358 | 13006195 |
| AT4G39470 | Chr4 | 18359373 | 18361170 | LOC_Os05g08930 | Chr5 | 4938258 | 4940959 | GSVIVG00019375001 | Chr7 | 12972511 | 12976928 |
| AT4G39520 | Chr4 | 18370968 | 18374204 | LOC_Os07g43470 | Chr7 | 26019539 | 26024756 | GSVIVG00019392001 | Chr7 | 12837681 | 12849561 |
| AT4G39620 | Chr4 | 18395294 | 18397578 | LOC_Os02g51480 | Chr2 | 31529599 | 31531859 | GSVIVG00019417001 | Chr7 | 12567937 | 12570510 |
| AT4G39660 | Chr4 | 18406765 | 18409564 | LOC_Os03g07570 | Chr3 | 3854304 | 3858411 | GSVIVG00019422001 | Chr7 | 12531489 | 12535746 |
| AT4G39670 | Chr4 | 18410172 | 18411000 | LOC_Os03g50280 | Chr3 | 28655240 | 28657434 | GSVIVG00030325001 | Chr1 | 3050062 | 3052892 |
| AT5G33370 | Chr5 | 12602468 | 12604764 | LOC_Os08g45150 | Chr8 | 28346373 | 28348793 | GSVIVG00026719001 | Chr4 | 9740602 | 9743482 |
| AT5G34940 | Chr5 | 13235829 | 13238839 | LOC_Os02g55870 | Chr2 | 34199419 | 34203705 | GSVIVG00006396001 | Chr11_random | 161176 | 164226 |
| AT5G35080 | Chr5 | 13354273 | 13356817 | LOC_Os06g43710 | Chr6 | 26316042 | 26318956 | GSVIVG00006387001 | Chr11_random | 1220 | 12961 |
| AT5G35100 | Chr5 | 13360283 | 13361390 | LOC_Os08g19610 | Chr8 | 11728223 | 11733120 | GSVIVG00010183001 | ChrUn_random | 145684536 | 145687443 |
| AT5G35160 | Chr5 | 13414751 | 13417205 | LOC_Os02g55440 | Chr2 | 33956291 | 33959749 | GSVIVG00005630001 | ChrUn_random | 33699557 | 33702038 |
| AT5G35170 | Chr5 | 13419166 | 13423746 | LOC_Os08g19140 | Chr8 | 11442523 | 11453895 | GSVIVG00005629001 | ChrUn_random | 33672581 | 33697322 |
| AT5G35180 | Chr5 | 13424418 | 13433018 | LOC_Os08g34060 | Chr8 | 21337593 | 21354581 | GSVIVG00005622001 | ChrUn_random | 33547914 | 33617766 |
| AT5G35220 | Chr5 | 13484431 | 13487584 | LOC_Os03g57840 | Chr3 | 32951544 | 32956889 | GSVIVG00003814001 | Chr8 | 2019329 | 2026169 |
| AT5G35320 | Chr5 | 13521779 | 13523425 | LOC_Os02g04860 | Chr2 | 2242926 | 2246169 | GSVIVG00007158001 | ChrUn_random | 45594883 | 45597206 |
| AT5G35330 | Chr5 | 13523475 | 13525787 | LOC_Os09g32090 | Chr9 | 19150469 | 19155881 | GSVIVG00004362001 | ChrUn_random | 92444709 | 92448817 |
| AT5G67140 | Chr5 | 26793880 | 26795303 | LOC_Os06g07000 | Chr6 | 3319111 | 3322612 | GSVIVG00019521001 | Chr7 | 11704578 | 11707765 |
| AT5G67170 | Chr5 | 26799766 | 26802067 | LOC_Os04g57480 | Chr4 | 34201019 | 34205682 | GSVIVG00019534001 | Chr7 | 11591227 | 11594750 |
| AT5G67270 | Chr5 | 26840070 | 26842081 | LOC_Os10g35580 | Chr10 | 19033885 | 19036321 | GSVIVG00019563001 | Chr7 | 11359290 | 11362393 |
| AT5G67300 | Chr5 | 26854022 | 26856006 | LOC_Os09g01960 | Chr9 | 656425 | 658374 | GSVIVG00019566001 | Chr7 | 11334164 | 11335506 |
| AT5G67320 | Chr5 | 26857154 | 26861119 | LOC_Os07g22220 | Chr7 | 12424060 | 12440486 | GSVIVG00019608001 | Chr7 | 11032914 | 11044816 |
| AT5G67360 | Chr5 | 26871891 | 26874579 | LOC_Os03g40830 | Chr3 | 22702919 | 22706164 | GSVIVG00019628001 | Chr7 | 10840878 | 10843432 |
| AT5G67470 | Chr5 | 26926713 | 26930470 | LOC_Os10g20710 | Chr10 | 10482035 | 10485936 | GSVIVG00019656001 | Chr7 | 10597220 | 10607612 |
| AT5G67530 | Chr5 | 26941182 | 26944146 | LOC_Os03g10400 | Chr3 | 5297334 | 5301353 | GSVIVG00012759001 | ChrUn_random | 151898481 | 151899236 |
| AT5G67570 | Chr5 | 26952325 | 26954847 | LOC_Os05g25060 | Chr5 | 14498742 | 14506826 | GSVIVG00004197001 | ChrUn_random | 90739624 | 90747202 |
| AT5G67590 | Chr5 | 26958001 | 26959557 | LOC_Os07g39710 | Chr7 | 23797954 | 23802656 | GSVIVG00010578001 | Chr7 | 10438094 | 10445150 |

**Table S4 Information of ten gene clusters in six plants (continued)**

| Brachy gene ID | Chr | Start | End | Maize gene ID | Chr | Start | End | Poplar gene ID | Chr | Start | End |
| --- | --- | --- | --- | --- | --- | --- | --- | --- | --- | --- | --- |
| Bradi1g04700 | Chr1 | 3147963 | 3148577 | GRMZM2G013342 | Chr1 | 20282072 | 20292395 | POPTR_0008s15100 | Chr 8 | 10092699 | 10093655 |
| Bradi1g24040 | Chr1 | 19300539 | 19304762 | GRMZM2G034651 | Chr 1 | 290448166 | 290449263 | POPTR_0005s23220 | Chr 5 | 22053690 | 22056035 |
| Bradi1g11700 | Chr1 | 8702376 | 8705250 | GRMZM2G123159 | Chr 10 | 131593783 | 131595368 | POPTR_0005s23200 | Chr 5 | 22037633 | 22040944 |
| Bradi2g26650 | Chr2 | 25387070 | 25394874 | GRMZM2G319109 | Chr 1 | 264164685 | 264167838 | POPTR_0005s23080 | Chr 5 | 21916547 | 21924034 |
| Bradi2g13200 | Chr2 | 11637776 | 11641307 | GRMZM2G701566 | Chr 6 | 143663383 | 143690661 | POPTR_0005s23030 | Chr 5 | 21871172 | 21873535 |
| Bradi1g78740 | Chr1 | 74635363 | 74640888 | GRMZM2G097605 | Chr 3 | 73963292 | 73983669 | POPTR_0002s05500 | Chr 2 | 3582384 | 3587533 |
| Bradi2g25850 | Chr2 | 24049180 | 24050505 | GRMZM2G323757 | Chr 10 | 89368914 | 89374442 | POPTR_0002s05580 | Chr 2 | 3651195 | 3652795 |
| Bradi4g42940 | Chr4 | 46789767 | 46792158 | GRMZM2G099052 | Chr 6 | 145492110 | 145493716 | POPTR_0019s06340 | Chr 19 | 7069259 | 7072864 |
| Bradi3g22380 | Chr3 | 21454895 | 21461560 | GRMZM2G064390 | Chr 8 | 65785879 | 65788921 | POPTR_0015s03060 | Chr 15 | 2567315 | 2575235 |
| Bradi2g26170 | Chr2 | 24503091 | 24505514 | GRMZM2G090905 | Chr 5 | 37076106 | 37099865 | POPTR_0002s22530 | Chr 2 | 19282884 | 19286028 |
| Bradi3g41190 | Chr3 | 43143582 | 43147183 | GRMZM2G123884 | Chr 6 | 144564725 | 144567105 | POPTR_0011s10450 | Chr 11 | 12887684 | 12891500 |
| Bradi3g08850 | Chr3 | 6952602 | 6954740 | GRMZM2G031311 | Chr 1 | 222601595 | 222603901 | POPTR_0001s39210 | Chr 1 | 38413028 | 38416564 |
| Bradi2g19640 | Chr2 | 17285105 | 17292225 | GRMZM2G068943 | Chr 6 | 133655707 | 133659307 | POPTR_0001s39220 | Chr 1 | 38422165 | 38433727 |
| Bradi4g28380 | Chr4 | 33787606 | 33790355 | GRMZM2G006678 | Chr 8 | 123601206 | 123609481 | POPTR_0011s10590 | Chr 11 | 13123625 | 13126082 |
| Bradi1g49260 | Chr1 | 48014684 | 48018866 | GRMZM2G070446 | Chr 2 | 176075354 | 176083102 | POPTR_0001s39260 | Chr 1 | 38473076 | 38477170 |
| Bradi2g49850 | Chr2 | 49855256 | 49857362 | GRMZM2G043212 | Chr 6 | 74457785 | 74463057 | POPTR_0001s39310 | Chr 1 | 38503646 | 38507882 |
| Bradi2g06430 | Chr2 | 4917163 | 4922794 | GRMZM2G435338 | Chr 3 | 197721783 | 197724923 | POPTR_0001s39560 | Chr 1 | 38772821 | 38776848 |
| Bradi5g25400 | Chr5 | 26740332 | 26743212 | GRMZM2G125083 | Chr 3 | 5428030 | 5436879 | POPTR_0011s14980 | Chr 11 | 17195249 | 17198454 |
| Bradi1g38180 | Chr1 | 34335267 | 34338028 | GRMZM5G812774 | Chr 10 | 147206883 | 147213776 | POPTR_0001s39520 | Chr 1 | 38731790 | 38735442 |
| Bradi1g68760 | Chr1 | 67271579 | 67280118 | GRMZM2G147603 | Chr 9 | 76462890 | 76466251 | POPTR_0001s39990 | Chr 1 | 39317875 | 39326129 |
| Bradi1g39720 | Chr1 | 36253562 | 36253996 | GRMZM2G101859 | Chr 1 | 31578024 | 31598791 | POPTR_0002s21870 | Chr 2 | 18562327 | 18563411 |
| Bradi4g33440 | Chr4 | 39119764 | 39122617 | GRMZM5G802566 | Chr 6 | 71732900 | 71733812 | POPTR_0014s15880 | Chr 14 | 11964262 | 11967624 |
| Bradi3g07140 | Chr3 | 5289890 | 5294406 | GRMZM2G007757 | Chr 7 | 131823217 | 131826370 | POPTR_0002s21840 | Chr 2 | 18535151 | 18540585 |
| Bradi1g42810 | Chr1 | 40186114 | 40190951 | GRMZM2G174170 | Chr 5 | 120574248 | 120578874 | POPTR_0014s15870 | Chr 14 | 11956648 | 11959970 |
| Bradi2g50040 | Chr2 | 49996381 | 50003394 | GRMZM2G176699 | Chr 6 | 120851002 | 120857801 | POPTR_0014s15850 | Chr 14 | 11942251 | 11945245 |
| Bradi1g35340 | Chr1 | 30833125 | 30837348 | GRMZM2G018105 | Chr 3 | 198597189 | 198606005 | POPTR_0002s21820 | Chr 2 | 18492852 | 18496268 |
| Bradi3g36130 | Chr3 | 38383843 | 38386287 | GRMZM2G116831 | Chr 6 | 97340072 | 97343799 | POPTR_0005s11240 | Chr 5 | 8144461 | 8147736 |
| Bradi1g15390 | Chr1 | 12324229 | 12326868 | GRMZM2G013600 | Chr 1 | 216299934 | 216302391 | POPTR_0012s13610 | Chr 12 | 13692122 | 13694273 |
| Bradi5g13780 | Chr5 | 17263268 | 17270861 | GRMZM2G168886 | Chr 1 | 246548207 | 246552407 | POPTR_0008s08910 | Chr 8 | 5517296 | 5524269 |
| Bradi3g00230 | Chr3 | 26069 | 27669 | GRMZM2G122793 | Chr 5 | 57186691 | 57203005 | POPTR_0001s34690 | Chr 1 | 32941898 | 32943995 |
| Bradi2g22400 | Chr2 | 19911770 | 19913585 | GRMZM2G039173 | Chr 9 | 9964958 | 9969714 | POPTR_0019s14730 | Chr 19 | 15405096 | 15406856 |
| Bradi1g63050 | Chr1 | 62260091 | 62264639 | GRMZM2G043948 | Chr 6 | 153623377 | 153625173 | POPTR_0013s15030 | Chr 13 | 15089469 | 15094236 |
| Bradi4g44480 | Chr4 | 47866517 | 47867451 | GRMZM2G042036 | Chr 1 | 56569008 | 56573612 | POPTR_0013s15260 | Chr 13 | 15290124 | 15292100 |
| Bradi2g53690 | Chr2 | 52804960 | 52806110 | GRMZM2G092517 | Chr 6 | 100205307 | 100214671 | POPTR_0013s08040 | Chr 13 | 6947839 | 6948785 |
| Bradi1g53730 | Chr1 | 52038333 | 52039875 | GRMZM2G138943 | Chr 3 | 184119671 | 184121779 | POPTR_0013s15190 | Chr 13 | 15208035 | 15210558 |
| Bradi2g62550 | Chr2 | 59143309 | 59148826 | GRMZM2G113325 | Chr 3 | 184213947 | 184216375 | POPTR_0019s00800 | Chr 19 | 534916 | 541150 |
| Bradi2g49650 | Chr2 | 49693892 | 49695833 | GRMZM2G143627 | Chr 7 | 172947746 | 172948399 | POPTR_0013s06850 | Chr 13 | 5536345 | 5539984 |
| Bradi2g31510 | Chr2 | 31266899 | 31270453 | GRMZM2G010468 | Chr 5 | 215652783 | 215654639 | POPTR_0013s15380 | Chr 13 | 15377403 | 15381827 |
| Bradi4g25900 | Chr4 | 31215555 | 31217868 | GRMZM2G353024 | Chr 2 | 151569232 | 151571445 | POPTR_0013s15500 | Chr 13 | 15448522 | 15451481 |
| Bradi2g31230 | Chr2 | 31046594 | 31049735 | GRMZM2G157705 | Chr 8 | 113211155 | 113229264 | POPTR_0006s11260 | Chr 6 | 8549290 | 8553107 |
| Bradi1g67750 | Chr1 | 66340473 | 66343392 | GRMZM2G013814 | Chr 8 | 170445762 | 170448153 | POPTR_0007s13790 | Chr 7 | 13829389 | 13832458 |
| Bradi5g25050 | Chr5 | 26509157 | 26510651 | GRMZM2G062396 | Chr 9 | 141835417 | 141842411 | POPTR_0005s11600 | Chr 5 | 8409877 | 8412181 |
| Bradi2g57330 | Chr2 | 55486411 | 55490331 | GRMZM2G038309 | Chr 2 | 3556786 | 3558729 | POPTR_0017s00430 | Chr 17 | 141255 | 146137 |
| Bradi4g36470 | Chr4 | 41692185 | 41694008 | GRMZM2G050268 | Chr 3 | 169743493 | 169748167 | POPTR_0005s11420 | Chr 5 | 8253399 | 8255315 |
| Bradi4g36480 | Chr4 | 41698115 | 41703557 | GRMZM2G071599 | Chr 2 | 196302759 | 196305162 | POPTR_0005s11440 | Chr 5 | 8266789 | 8269881 |
| Bradi3g51190 | Chr3 | 52288459 | 52294645 | GRMZM2G080644 | Chr 7 | 176021673 | 176025491 | POPTR_0005s10680 | Chr 5 | 7676775 | 7683162 |
| Bradi5g19560 | Chr5 | 22579502 | 22583114 | GRMZM2G066489 | Chr 4 | 154422649 | 154429437 | POPTR_0002s00870 | Chr 2 | 401985 | 407592 |
| Bradi2g21500 | Chr2 | 18981784 | 18982536 | GRMZM2G117558 | Chr 2 | 14397636 | 14401986 | POPTR_0001s46010 | Chr 1 | 46523907 | 46525488 |
| Bradi1g42650 | Chr1 | 39972663 | 39975622 | GRMZM2G042356 | Chr 6 | 155508807 | 155509893 | POPTR_0011s14890 | Chr 11 | 17138884 | 17142785 |
| Bradi2g04110 | Chr2 | 2882961 | 2884855 | GRMZM5G877259 | Chr 4 | 162054688 | 162058019 | POPTR_0006s10790 | Chr 6 | 8155568 | 8160931 |
| Bradi1g70410 | Chr1 | 68749913 | 68760124 | GRMZM2G002002 | Chr 3 | 13686214 | 13691557 | POPTR_0002s17090 | Chr 2 | 12867486 | 12875604 |
| Bradi3g16950 | Chr3 | 15102696 | 15105487 | GRMZM2G077486 | Chr 10 | 80405919 | 80409657 | POPTR_0002s18450 | Chr 2 | 14365586 | 14369660 |
| Bradi2g11090 | Chr2 | 9280270 | 9284625 | GRMZM2G115875 | Chr 3 | 48793405 | 48804206 | POPTR_0002s18720 | Chr 2 | 14584400 | 14587508 |
| Bradi1g02720 | Chr1 | 1829154 | 1832194 | GRMZM2G057296 | Chr 7 | 24405653 | 24409093 | POPTR_0002s18800 | Chr 2 | 14681515 | 14685081 |
| Bradi4g07850 | Chr4 | 6758409 | 6770150 | GRMZM2G094123 | Chr 4 | 215212969 | 215240759 | POPTR_0002s18810 | Chr 2 | 14702697 | 14707800 |
| Bradi2g12100 | Chr2 | 10445668 | 10449341 | GRMZM2G097103 | Chr 8 | 38489499 | 38493433 | POPTR_0014s10850 | Chr 14 | 8100146 | 8102531 |
| Bradi1g20130 | Chr1 | 16122450 | 16125453 | GRMZM2G070588 | Chr 7 | 168570972 | 168572448 | POPTR_0005s00580 | Chr 5 | 233302 | 237371 |
| Bradi2g31550 | Chr2 | 31357810 | 31362505 | GRMZM2G432566 | Chr 1 | 20213131 | 20219312 | POPTR_0002s19040 | Chr 2 | 14939062 | 14943880 |
| Bradi2g11350 | Chr2 | 9605794 | 9607897 | GRMZM2G060798 | Chr 10 | 85581746 | 85584681 | POPTR_0002s19100 | Chr 2 | 15007069 | 15009907 |
| Bradi4g04420 | Chr4 | 3608554 | 3612498 | GRMZM2G082203 | Chr 3 | 128093127 | 128097800 | POPTR_0002s19170 | Chr 2 | 15089512 | 15093802 |
| Bradi1g45370 | Chr1 | 43578535 | 43583796 | GRMZM2G067373 | Chr 6 | 112349351 | 112360258 | POPTR_0002s09180 | Chr 2 | 6499367 | 6504325 |
| Bradi4g06800 | Chr4 | 5694100 | 5697753 | GRMZM2G145578 | Chr 10 | 17164898 | 17170671 | POPTR_0008s04680 | Chr 8 | 2681655 | 2685427 |
| Bradi1g20330 | Chr1 | 16298220 | 16299992 | GRMZM2G099207 | Chr 7 | 168005392 | 168007659 | POPTR_0001s04270 | Chr 1 | 3375235 | 3377009 |
| Bradi5g02160 | Chr5 | 2252172 | 2255407 | GRMZM2G174671 | Chr 10 | 94095402 | 94103354 | POPTR_0002s14720 | Chr 2 | 10929894 | 10932398 |
| Bradi3g46590 | Chr3 | 48404949 | 48407810 | GRMZM2G024739 | Chr 5 | 175728635 | 175732381 | POPTR_0002s09730 | Chr 2 | 6972896 | 6977306 |
| Bradi1g45610 | Chr1 | 43877265 | 43881893 | GRMZM2G058560 | Chr 9 | 6263413 | 6267851 | POPTR_0002s09920 | Chr 2 | 7109701 | 7110927 |
| Bradi1g45630 | Chr1 | 43899318 | 43901170 | GRMZM5G801076 | Chr 5 | 208740969 | 208744027 | POPTR_0002s09930 | Chr 2 | 7120289 | 7123581 |
| Bradi2g61370 | Chr2 | 58483860 | 58490164 | GRMZM2G133952 | Chr 8 | 157401584 | 157408704 | POPTR_0019s10740 | Chr 19 | 12257494 | 12269680 |
| Bradi1g27120 | Chr1 | 22218008 | 22220042 | GRMZM2G134797 | Chr 7 | 149915725 | 149918129 | POPTR_0002s13970 | Chr 2 | 10357071 | 10358610 |
| Bradi4g24390 | Chr4 | 29529571 | 29532964 | GRMZM2G035843 | Chr 4 | 204664489 | 204668662 | POPTR_0013s11690 | Chr 13 | 12351075 | 12356757 |
| Bradi2g09540 | Chr2 | 7803480 | 7806096 | GRMZM2G029731 | Chr 8 | 4615830 | 4619658 | POPTR_0009s12340 | Chr 9 | 10076335 | 10079273 |
| Bradi3g58130 | Chr3 | 57685815 | 57690939 | GRMZM2G101463 | Chr 5 | 209693887 | 209699045 | POPTR_0004s16240 | Chr 4 | 16639425 | 16647499 |
| Bradi5g10120 | Chr5 | 13373995 | 13378597 | GRMZM2G147667 | Chr 2 | 51815271 | 51819205 | POPTR_0007s07040 | Chr 7 | 5368335 | 5374209 |
| Bradi2g48280 | Chr2 | 48579490 | 48582858 | GRMZM2G048294 | Chr 8 | 155077128 | 155081200 | POPTR_0005s08880 | Chr 5 | 6200573 | 6204553 |
| Bradi1g01770 | Chr1 | 1169060 | 1172470 | GRMZM2G042027 | Chr 5 | 204612183 | 204628810 | POPTR_0005s08790 | Chr 5 | 6126510 | 6131173 |
| Bradi2g33620 | Chr2 | 33741326 | 33745949 | GRMZM5G847615 | Chr 6 | 127893190 | 127895627 | POPTR_0005s08770 | Chr 5 | 6114422 | 6118112 |
| Bradi1g20620 | Chr1 | 16491585 | 16496185 | GRMZM2G158021 | Chr 7 | 167333820 | 167340329 | POPTR_0007s06600 | Chr 7 | 5015036 | 5022058 |
| Bradi3g59120 | Chr3 | 58366536 | 58368264 | GRMZM2G025409 | Chr 4 | 181425916 | 181428451 | POPTR_0005s08410 | Chr 5 | 5809761 | 5811595 |
| Bradi1g73120 | Chr1 | 70725771 | 70729142 | GRMZM2G124353 | Chr 1 | 15353246 | 15356638 | POPTR_0007s06190 | Chr 7 | 4354307 | 4358371 |
| Bradi1g11280 | Chr1 | 8332583 | 8333953 | GRMZM2G011098 | Chr 5 | 11390627 | 11392724 | POPTR_0008s11830 | Chr 8 | 7575506 | 7577980 |
| Bradi3g12730 | Chr3 | 11423331 | 11424458 | GRMZM2G148867 | Chr 1 | 201738864 | 201745838 | POPTR_0013s04780 | Chr 13 | 3294496 | 3297506 |
| Bradi3g54370 | Chr3 | 54850159 | 54853462 | GRMZM2G083812 | Chr 5 | 214166462 | 214170119 | POPTR_0006s06100 | Chr 6 | 4336722 | 4340025 |
| Bradi1g30600 | Chr1 | 25914149 | 25916454 | GRMZM2G044354 | Chr 1 | 147343802 | 147353390 | POPTR_0006s06030 | Chr 6 | 4284553 | 4288842 |
| Bradi3g19920 | Chr3 | 18911621 | 18917326 | GRMZM2G085885 | Chr 1 | 2741608 | 2750630 | POPTR_0006s20430 | Chr 6 | 19443299 | 19446938 |
| Bradi3g54220 | Chr3 | 54707794 | 54709770 | GRMZM2G469111 | Chr 5 | 213944858 | 213947602 | POPTR_0006s20360 | Chr 6 | 19406744 | 19409454 |
| Bradi3g19850 | Chr3 | 18831944 | 18842258 | GRMZM2G030628 | Chr 4 | 66899011 | 66909708 | POPTR_0018s12140 | Chr 18 | 12715057 | 12721558 |
| Bradi3g37010 | Chr3 | 39322614 | 39339649 | GRMZM2G160927 | Chr 1 | 212287664 | 212305641 | POPTR_0018s12110 | Chr 18 | 12684392 | 12700594 |
| Bradi1g06080 | Chr1 | 4085788 | 4092303 | GRMZM2G007907 | Chr 5 | 4502664 | 4517703 | POPTR_0013s06090 | Chr 13 | 4498137 | 4503617 |
| Bradi3g03630 | Chr3 | 2409693 | 2412733 | GRMZM2G117459 | Chr 4 | 237692856 | 237696509 | POPTR_0006s07020 | Chr 6 | 5063788 | 5065180 |
| Bradi4g34050 | Chr4 | 39703402 | 39708576 | GRMZM2G025095 | Chr 2 | 190664560 | 190686734 | POPTR_0006s07730 | Chr 6 | 5633288 | 5637730 |
| Bradi1g48360 | Chr1 | 47004602 | 47007104 | GRMZM2G481291 | Chr 9 | 16286452 | 16295036 | POPTR_0005s18320 | Chr 5 | 16472184 | 16474519 |
| Bradi5g25650 | Chr5 | 26863985 | 26868110 | GRMZM2G107187 | Chr 2 | 2533374 | 2536615 | POPTR_0005s18390 | Chr 5 | 16556509 | 16561504 |
| Bradi3g30010 | Chr3 | 32038458 | 32040688 | GRMZM2G045808 | Chr 1 | 227056986 | 227059071 | POPTR_0005s18580 | Chr 5 | 16814354 | 16816462 |
| Bradi4g08220 | Chr4 | 7202041 | 7203051 | GRMZM2G050550 | Chr 7 | 51469024 | 51471534 | POPTR_0002s12410 | Chr 2 | 9199640 | 9200728 |
| Bradi1g52640 | Chr1 | 50944488 | 50953976 | GRMZM2G159330 | Chr 10 | 101031429 | 101049182 | POPTR_0007s10380 | Chr 7 | 10301016 | 10307955 |
| Bradi1g14860 | Chr1 | 11820810 | 11823119 | GRMZM2G121293 | Chr 1 | 249535580 | 249538534 | POPTR_0002s12130 | Chr 2 | 9002031 | 9004755 |
| Bradi1g70900 | Chr1 | 69026975 | 69030652 | GRMZM2G075921 | Chr 9 | 147732752 | 147737234 | POPTR_0007s09870 | Chr 7 | 9392080 | 9399932 |
| Bradi1g71100 | Chr1 | 69193321 | 69196734 | GRMZM2G007486 | Chr 1 | 22736088 | 22739715 | POPTR_0005s19290 | Chr 5 | 18008265 | 18012911 |
| Bradi1g15900 | Chr1 | 12841815 | 12848591 | GRMZM5G884466 | Chr 1 | 301264981 | 301284057 | POPTR_0007s14930 | Chr 7 | 14609148 | 14614098 |
| Bradi1g23160 | Chr1 | 18572734 | 18578682 | GRMZM2G396397 | Chr 7 | 161685025 | 161693219 | POPTR_0002s11860 | Chr 2 | 8798154 | 8800706 |
