# Supplementary material for: Evaluation of Genome Sequencing Quality in Selected Plant Species Using Expressed Sequence Tags
Source: PLoS One. 2013 Jul 29;8(7):e69890. doi: 10.1371/journal.pone.0069890 (PMC3726750; doi:10.1371/journal.pone.0069890)
Supplement: Table S5 — Information of homologue genes involved in anthocyanin biosynthesis pathway in three plants. (DOC) [file pone.0069890.s007.doc]

**Table S5 Information of homologue genes involved in anthocyanin biosynthesis pathway in three plants**

| Arabidopsis gene | Gene ID | Chr | Start | End | Maize gene | Gene ID | Chr | Start | End | Grape gene | Gene ID | Chr | Start | End |
| --- | --- | --- | --- | --- | --- | --- | --- | --- | --- | --- | --- | --- | --- | --- |
| *AtPAL1* | AT2G37040 | 2 | 15557376 | 15560363 | *ZmPAL1* | GRMZM2G118345 | 2 | 28048958 | 28052061 | *VvPAL1* | GSVIVP00013939001 | 16 | 700746 | 703220 |
| *AtPAL2* | AT3G53260 | 3 | 19744051 | 19746780 | *ZmPAL2* | GRMZM2G170692 | 5 | 186795550 | 186798126 | *VvPAL2* | GSVIVP00013930001 | 16 | 648662 | 651184 |
| *AtPAL4* | AT3G10340 | 3 | 3204013 | 3208022 | *ZmPAL3* | GRMZM2G081582 | 4 | 143464625 | 143469700 | *VvPAL3* | GSVIVP00013943001 | 16 | 724118 | 727239 |
| *AtPAL3* | AT5G04230 | 5 | 1160831 | 1163775 | *ZmPAL4* | GRMZM2G334660 | 5 | 186732964 | 186735665 | *VvPAL4* | GSVIVP00013928001 | 16 | 618476 | 621190 |
|  |  |  |  |  | *ZmPAL5* | GRMZM2G029048 | 5 | 186726200 | 186730209 | *VvPAL5* | GSVIVP00013947001 | 16 | 779795 | 782254 |
|  |  |  |  |  | *ZmPAL6* | GRMZM2G063917 | 4 | 143512171 | 143515157 | *VvPAL6* | GSVIVP00013922001 | 16 | 597226 | 599843 |
|  |  |  |  |  | *ZmPAL7* | GRMZM2G441347 | 2 | 28117445 | 28120590 | *VvPAL7* | GSVIVP00023211001 | 8 | 11718432 | 11721087 |
|  |  |  |  |  | *ZmPAL8* | GRMZM2G074604 | 5 | 186677004 | 186680745 | *VvPAL8* | GSVIVP00018175001 | 13 | 5969524 | 5991084 |
|  |  |  |  |  |  |  |  |  |  | *VvPAL9* | GSVIVP00024561001 | 6 | 3222841 | 3226347 |
|  |  |  |  |  |  |  |  |  |  | *VvPAL10* | GSVIVP00013927001 | 16 | 612547 | 614853 |
|  |  |  |  |  |  |  |  |  |  | *VvPAL11* | GSVIVP00013924001 | 16 | 606754 | 608798 |
| *AtC4H* | AT2G30490 | 2 | 12993663 | 12995770 | *ZmC4H1* | GRMZM2G010468 | 8 | 170445762 | 170448153 | *VvC4H1* | GSVIVP00023932001 | 6 | 8813071 | 8816574 |
|  |  |  |  |  | *ZmC4H2* | GRMZM2G139874 | 8 | 83792802 | 83796118 | *VvC4H2* | GSVIVP00017017001 | 11 | 10158634 | 10160529 |
|  |  |  |  |  |  |  |  |  |  | *VvC4H3* | GSVIVP00007155001 | Un_random | 45548882 | 45550610 |
| *At4CL1* | AT1G51680 | 1 | 19158752 | 19161552 | *Zm4CL1* | GRMZM2G075333 | 5 | 89152269 | 89156855 | *Vv4CL1* | GSVIVP00031383001 | 11 | 12980141 | 12983730 |
| *At4CL2* | AT3G21240 | 3 | 7454269 | 7457379 | *Zm4CL2* | GRMZM2G055320 | 9 | 107790531 | 107797125 | *Vv4CL2* | GSVIVP00031385001 | 11 | 13004990 | 13010232 |
| *At4CL3* | AT1G65060 | 1 | 24167202 | 24171502 | *Zm4CL3* | GRMZM2G174732 | 4 | 71907725 | 71918757 | *Vv4CL3* | GSVIVP00014031001 | 16 | 2123569 | 2128535 |
| *At4CL4* | AT5G63380 | 5 | 25387411 | 25390063 | *Zm4CL4* | GRMZM2G122787 | 1 | 11528522 | 11534033 | *Vv4CL4* | GSVIVP00022179001 | 6 | 22071551 | 22079236 |
| *At4CL5* | AT3G21230 | 3 | 7448040 | 7452000 | *Zm4CL5* | GRMZM2G014651 | 2 | 53200444 | 53202745 | *Vv4CL5* | GSVIVP00002799001 | 1_random | 4850822 | 4853094 |
| *At4CL6* | AT4G05160 | 4 | 2664385 | 2666707 | *Zm4CL6* | GRMZM2G145179 | 3 | 2343134 | 2345435 | *Vv4CL6* | GSVIVP00018101001 | 13 | 7219492 | 7221432 |
| *At4CL7* | AT4G19010 | 4 | 10411490 | 10414249 | *Zm4CL7* | GRMZM2G174574 | 1 | 245027693 | 245032711 | *Vv4CL7* | GSVIVP00015553001 | 18 | 1226323 | 1230351 |
| *At4CL8* | AT5G38120 | 5 | 15213765 | 15216205 | *Zm4CL8* | GRMZM2G054013 | 4 | 158142358 | 158147479 | *Vv4CL8* | GSVIVP00003591001 | Un_random | 26173966 | 26179603 |
| *At4CL9* | AT1G20510 | 1 | 7103454 | 7105881 | *Zm4CL9* | GRMZM2G096020 | 1 | 7341979 | 7348599 | *Vv4CL9* | GSVIVP00020555001 | 14 | 15331127 | 15333537 |
| *At4CL10* | AT1G62940 | 1 | 23310535 | 23312747 |  |  |  |  |  | *Vv4CL10* | GSVIVP00020554001 | 14 | 15323828 | 15330405 |
| *At4CL12* | AT1G20500 | 1 | 7100502 | 7102915 |  |  |  |  |  |  |  |  |  |  |
| *At4CL14* | AT1G20480 | 1 | 7094833 | 7097114 |  |  |  |  |  |  |  |  |  |  |
| *AtCHS* | AT5G13930 | 5 | 4488688 | 4490264 | *ZmCHS* | GRMZM2G151227 | 2 | 223888706 | 223892691 | *VvCHS1* | GSVIVP00037969001 | 14 | 13875792 | 13877198 |
|  |  |  |  |  |  |  |  |  |  | *VvCHS2* | GSVIVP00037967001 | 14 | 13889324 | 13890882 |
|  |  |  |  |  |  |  |  |  |  | *VvCHS3* | GSVIVP00006341001 | Un_random | 37798575 | 37800731 |
| *AtCHI* | AT3G55120 | 3 | 20430114 | 20431470 | *ZmCHI* | GRMZM2G155329 | 1 | 293041177 | 293042705 | *VvCHI* | GSVIVP00029513001 | 13 | 2128014 | 2129693 |
| *AtF3H* | AT3G51240 | 3 | 19025264 | 19026939 | *ZmF3H* | GRMZM2G062396 | 2 | 3556786 | 3558729 | *VvF3H1* | GSVIVP00036784001 | 4 | 14790912 | 14792727 |
|  |  |  |  |  |  |  |  |  |  | *VvF3H2* | GSVIVP00014419001 | 18 | 12160108 | 12162146 |
|  |  |  |  |  |  |  |  |  |  | *VvF3H3* | GSVIVP00036782001 | 4 | 14832092 | 14835848 |
| *AtF3'H* | AT5G07990 | 5 | 2560394 | 2563109 | *ZmF3'H1* | GRMZM2G025832 | 5 | 180038179 | 180040249 | *VvF3'H1* | GSVIVP00016217001 | 17 | 8112032 | 8114427 |
|  |  |  |  |  | *ZmF3'H2* | GRMZM2G313750 | 4 | 130236777 | 130246440 | *VvF3'H2* | GSVIVP00016215001 | 17 | 8135555 | 8137842 |
| *AtF3'5'H1* | AT4G12300 | 4 | 7307737 | 7309755 | *ZmF3'5'H1* | GRMZM2G089528 | 1 | 207274164 | 207276641 | *VvF3'5'H1* | GSVIVP00007272001 | 2 | 4774838 | 4777086 |
| *AtF3'5'H2* | AT4G12320 | 4 | 7314778 | 7316669 | *ZmF3'5'H2* | GRMZM2G158342 | 3 | 13101531 | 13103292 | *VvF3'5'H2* | GSVIVP00007269001 | 2 | 4754383 | 4756418 |
| *AtF3'5'H3* | AT4G12310 | 4 | 7310416 | 7312577 | *ZmF3'5'H3* | GRMZM2G049424 | 4 | 140890485 | 140895190 | *VvF3'5'H3* | GSVIVP00010339001 | Un_random | 63649713 | 63651747 |
| *AtF3'5'H4* | AT4G12330 | 4 | 7317560 | 7319739 | *ZmF3'5'H4* | GRMZM2G160763 | 8 | 116203880 | 116208585 | *VvF3'5'H4* | GSVIVP00007267001 | 2 | 4745715 | 4747542 |
| *AtF3'5'H5* | AT5G44620 | 5 | 17997779 | 17999558 | *ZmF3'5'H5* | GRMZM2G321033 | 1 | 33952766 | 33956487 | *VvF3'5'H5* | GSVIVP00010337001 | Un_random | 63642459 | 63644286 |
| *AtF3'5'H6* | AT4G22690 | 4 | 11929359 | 11931693 | *ZmF3'5'H6* | GRMZM5G851862 | 5 | 21341590 | 21344531 | *VvF3'5'H6* | GSVIVP00010332001 | Un_random | 63611190 | 63613352 |
| *AtF3'5'H7* | AT4G22710 | 4 | 11934969 | 11936755 |  |  |  |  |  | *VvF3'5'H7* | GSVIVP00010336001 | Un_random | 63635888 | 63637834 |
|  |  |  |  |  |  |  |  |  |  | *VvF3'5'H8* | GSVIVP00007266001 | 2 | 4738982 | 4741051 |
|  |  |  |  |  |  |  |  |  |  | *VvF3'5'H9* | GSVIVP00038447001 | 16_random | 1531571 | 1533743 |
|  |  |  |  |  |  |  |  |  |  | *VvF3'5'H10* | GSVIVP00038441001 | 16_random | 1444657 | 1446826 |
|  |  |  |  |  |  |  |  |  |  | *VvF3'5'H11* | GSVIVP00038443001 | 16_random | 1473707 | 1475916 |
|  |  |  |  |  |  |  |  |  |  | *VvF3'5'H12* | GSVIVP00001045001 | 2 | 4336836 | 4339311 |
|  |  |  |  |  |  |  |  |  |  | *VvF3'5'H13* | GSVIVP00026276001 | 15 | 6313161 | 6315563 |
|  |  |  |  |  |  |  |  |  |  | *VvF3'5'H14* | GSVIVP00015660001 | 2 | 9317991 | 9320950 |
|  |  |  |  |  |  |  |  |  |  | *VvF3'5'H15* | GSVIVP00001048001 | 2 | 4317518 | 4319383 |
|  |  |  |  |  |  |  |  |  |  | *VvF3'5'H16* | GSVIVP00026282001 | 15 | 6260387 | 6262922 |
|  |  |  |  |  |  |  |  |  |  | *VvF3'5'H17* | GSVIVP00022287001 | 6 | 20475674 | 20477909 |
|  |  |  |  |  |  |  |  |  |  | *VvF3'5'H18* | GSVIVP00005727001 | 3 | 9985993 | 9987872 |
|  |  |  |  |  |  |  |  |  |  | *VvF3'5'H19* | GSVIVP00026277001 | 15 | 6309081 | 6311743 |
|  |  |  |  |  |  |  |  |  |  | *VvF3'5'H20* | GSVIVP00016437001 | 11 | 832958 | 835061 |
|  |  |  |  |  |  |  |  |  |  | *VvF3'5'H21* | GSVIVP00003596001 | Un_random | 26288189 | 26290502 |
|  |  |  |  |  |  |  |  |  |  | *VvF3'5'H22* | GSVIVP00015613001 | 2 | 10830659 | 10833635 |
|  |  |  |  |  |  |  |  |  |  | *VvF3'5'H23* | GSVIVP00016433001 | 11 | 811943 | 813571 |
|  |  |  |  |  |  |  |  |  |  | *VvF3'5'H24* | GSVIVP00015616001 | 2 | 10779040 | 10785958 |
|  |  |  |  |  |  |  |  |  |  | *VvF3'5'H25* | GSVIVP00026289001 | 15 | 6166643 | 6169581 |
|  |  |  |  |  |  |  |  |  |  | *VvF3'5'H26* | GSVIVP00034070001 | 9 | 824462 | 826981 |
|  |  |  |  |  |  |  |  |  |  | *VvF3'5'H27* | GSVIVP00021704001 | 8 | 17059699 | 17061345 |
|  |  |  |  |  |  |  |  |  |  | *VvF3'5'H28* | GSVIVP00026288001 | 15 | 6180928 | 6183234 |
|  |  |  |  |  |  |  |  |  |  | *VvF3'5'H29* | GSVIVP00017654001 | 17 | 3820820 | 3823244 |
|  |  |  |  |  |  |  |  |  |  | *VvF3'5'H30* | GSVIVP00025511001 | 8 | 20193957 | 20195681 |
| *AtDFR* | AT5G42800 | 5 | 17164141 | 17165918 | *ZmDFR1* | GRMZM2G013726 | 8 | 146110828 | 146112451 | *VvDFR* | GSVIVP00014584001 | 18 | 10768605 | 10771679 |
|  |  |  |  |  | *ZmDFR2* | GRMZM2G026930 | 3 | 216304734 | 216306568 |  |  |  |  |  |
| *AtANS/AtLDOX* | AT4G22880 | 4 | 12004768 | 12006209 | *ZmANS/AtLDOX* | GRMZM2G345717 | 5 | 66107721 | 66110559 | *VvANS/AtLDOX* | GSVIVP00001063001 | 2 | 4220998 | 4222539 |
| *AtUFGT1* | AT5G17050 | 5 | 5607788 | 5609492 | *ZmUFGT1* | GRMZM2G165390 | 9 | 11774733 | 11776491 | *VvUFGT* | GSVIVP00014047001 | 16 | 2334697 | 2341263 |
| *AtUFGT2* | AT5G17030 | 5 | 5603133 | 5604738 | *ZmUFGT2* | GRMZM2G022242 | 2 | 207384991 | 207386776 |  |  |  |  |  |
| *AtUFGT3* | AT1G30530 | 1 | 10814659 | 10816583 |  |  |  |  |  |  |  |  |  |  |
| *AtUFGT4* | AT5G17040 | 5 | 5605284 | 5606970 |  |  |  |  |  |  |  |  |  |  |
